# Partial Inhibition of Adipose Tissue Lipolysis Improves Glucose Metabolism and Insulin Sensitivity Without Alteration of Fat Mass

**DOI:** 10.1371/journal.pbio.1001485

**Published:** 2013-02-19

**Authors:** Amandine Girousse, Geneviève Tavernier, Carine Valle, Cedric Moro, Niklas Mejhert, Anne-Laure Dinel, Marianne Houssier, Balbine Roussel, Aurèle Besse-Patin, Marion Combes, Lucile Mir, Laurent Monbrun, Véronic Bézaire, Bénédicte Prunet-Marcassus, Aurélie Waget, Isabelle Vila, Sylvie Caspar-Bauguil, Katie Louche, Marie-Adeline Marques, Aline Mairal, Marie-Laure Renoud, Jean Galitzky, Cecilia Holm, Etienne Mouisel, Claire Thalamas, Nathalie Viguerie, Thierry Sulpice, Rémy Burcelin, Peter Arner, Dominique Langin

**Affiliations:** 1 INSERM, UMR1048, Obesity Research Laboratory, Institute of Metabolic and Cardiovascular Diseases, Toulouse, France; 2 University of Toulouse, UMR1048, Paul Sabatier University, France; 3 Department of Medicine, Karolinska Institute at Karolinska Hospital, Huddinge, Stockholm, Sweden; 4 Physiogenex, Prologue Biotech, Labège-Innopole, France; 5 INSERM, UMR1048,Team 2, I2MC, Institute of Metabolic and Cardiovascular Diseases, Toulouse, France; 6 Toulouse University Hospitals, Laboratory of Clinical Biochemistry, Toulouse, France; 7 INSERM, UMR1048, Team 1, I2MC, Institute of Metabolic and Cardiovascular Diseases, Toulouse, France; 8 Department of Experimental Medical Science, Lund University, Lund, Sweden; 9 Toulouse University Hospitals, INSERM, Clinical Investigation Center, Toulouse, France; University of Cambridge, United Kingdom

## Abstract

When energy is needed, white adipose tissue (WAT) provides fatty acids (FAs) for use in peripheral tissues via stimulation of fat cell lipolysis. FAs have been postulated to play a critical role in the development of obesity-induced insulin resistance, a major risk factor for diabetes and cardiovascular disease. However, whether and how chronic inhibition of fat mobilization from WAT modulates insulin sensitivity remains elusive. Hormone-sensitive lipase (HSL) participates in the breakdown of WAT triacylglycerol into FAs. HSL haploinsufficiency and treatment with a HSL inhibitor resulted in improvement of insulin tolerance without impact on body weight, fat mass, and WAT inflammation in high-fat-diet–fed mice. In vivo palmitate turnover analysis revealed that blunted lipolytic capacity is associated with diminution in FA uptake and storage in peripheral tissues of obese HSL haploinsufficient mice. The reduction in FA turnover was accompanied by an improvement of glucose metabolism with a shift in respiratory quotient, increase of glucose uptake in WAT and skeletal muscle, and enhancement of de novo lipogenesis and insulin signalling in liver. In human adipocytes, HSL gene silencing led to improved insulin-stimulated glucose uptake, resulting in increased de novo lipogenesis and activation of cognate gene expression. In clinical studies, WAT lipolytic rate was positively and negatively correlated with indexes of insulin resistance and WAT de novo lipogenesis gene expression, respectively. In obese individuals, chronic inhibition of lipolysis resulted in induction of WAT de novo lipogenesis gene expression. Thus, reduction in WAT lipolysis reshapes FA fluxes without increase of fat mass and improves glucose metabolism through cell-autonomous induction of fat cell de novo lipogenesis, which contributes to improved insulin sensitivity.

## Abstract 2

Partial inhibition of adipose tissue lipolysis does not increase fat mass but improves glucose metabolism and insulin sensitivity through modulation of fatty acid turnover and induction of fat cell de novo lipogenesis.

## Author Summary

In periods of energy demand, mobilization of fat stores in mammals (i.e., adipose tissue lipolysis) is essential to provide energy in the form of fatty acids. In excess, however, fatty acids induce resistance to the action of insulin, which serves to regulate glucose metabolism in skeletal muscle and liver. Insulin resistance (or low insulin sensitivity) is believed to be a cornerstone of the complications of obesity such as type 2 diabetes and cardiovascular diseases. In this study, our clinical observation of natural variation in fat cell lipolysis in individuals reveals that a high lipolytic rate is associated with low insulin sensitivity. Furthermore, partial genetic and pharmacologic inhibition of hormone-sensitive lipase, one of the enzymes involved in the breakdown of white adipose tissue lipids, results in improvement of insulin sensitivity in mice without gain in body weight and fat mass. We undertake a series of mechanistic studies in mice and in human fat cells to show that blunted lipolytic capacity increases the synthesis of new fatty acids from glucose in fat cells, a pathway that has recently been shown by others to be a major determinant of whole body insulin sensitivity. In conclusion, partial inhibition of adipose tissue lipolysis is a plausible strategy in the treatment of obesity-related insulin resistance.

## Introduction

White adipose tissue (WAT) is the main energy store of the body in mammals. In the fed state, under the influence of insulin, WAT stores excess energy as triacylglycerols (TGs) in the lipid droplet of adipocytes. When energy is needed between meals or during physical exercise, WAT delivers fatty acids (FAs) to be oxidized in peripheral tissues. Lipolysis is the process by which stored TGs are released as nonesterified FA (NEFA) [1]. It involves different regulators such as lipases, co-lipases, and proteins that coat the lipid droplet. It is now largely accepted that the enzymatic breakdown of TG is initiated by adipose triglyceride lipase (ATGL) and leads to the formation of diacylglycerols (DGs) that are in turn hydrolyzed by hormone-sensitive lipase (HSL) [2],[3]. HSL also shows TG hydrolase activity. The final step of this catabolic process is the hydrolysis of monoacylglycerols by monoglyceride lipase, leading to the release of one molecule of glycerol and three molecules of FA.

Obese individuals are at increased risk of type 2 diabetes and cardiovascular disease. Insulin resistance is viewed as a cornerstone of the underlying pathogenic processes, and there is a wide disparity in insulin resistance among obese individuals [4]. FAs have been postulated to play a critical role in the development of insulin resistance [5]. Plasma NEFA levels and fluxes are not directly determined by the amount of body fat and are partly controlled by WAT lipolysis [6]. Furthermore, the relationship between circulating NEFA concentrations and insulin sensitivity in vivo is not straight forward. In that context, the influence of variation in fat cell lipolysis and lipase expression on insulin sensitivity and glucose metabolism remains elusive. Different consequences of diminished WAT lipolysis that are not mutually exclusive can be hypothesized. It could favour the development of obesity through retention of TG within adipocytes. It can also be viewed as a mechanism limiting an excess of FA release and alleviating the development of insulin resistance and metabolic abnormalities. This effect may be direct through the deleterious action of FA on insulin-sensitive tissues [7]. An indirect action may be considered via the modulation of WAT inflammation-induced insulin resistance as FAs produced by adipocytes stimulate cytokine production by macrophages [8]. Moreover, there is no clear picture of the relation between WAT lipolysis and glucose metabolism in WAT, skeletal muscle, and liver. Mice with full knockout of HSL and ATGL show complex phenotypes including marked alterations of fat mass that preclude conclusions on the effect of WAT lipolysis inhibition on insulin sensitivity [9]. Therefore, a major question of clinical relevance that remains unanswered is the role of FA release from WAT on insulin sensitivity and glucose metabolism and whether this control operates with an effect on fat mass.

By a combination of clinical, animal, and cellular studies, we investigated the role of WAT lipolysis, including the specific contribution of HSL, in the control of insulin sensitivity and glucose metabolism. HSL level is a determinant of lipolytic capacity in human WAT [10]. Reduction of HSL activity and blunted stimulated lipolysis has been observed in obesity [11],[12]. To gain mechanistic insights in this relationship, we investigated HSL haploinsufficient (HSL^+/−^) mice that showed impaired HSL expression and enzymatic activity and diminished WAT lipolysis. When fed a high-fat diet (HFD), HSL^+/−^ mice did not become more obese than wild type (WT) littermates and their WAT was not more inflamed. Lipid metabolism studied dynamically in vivo revealed a global reduction of FA fluxes in HSL^+/−^ mice. The slowdown of FA turnover was accompanied by an amelioration of glucose metabolism, including glucose uptake and de novo lipogenesis in insulin-sensitive tissues. Insulin tolerance was improved both in mice with HSL haploinsufficiency and in mice treated with a HSL inhibitor. Improved adipocyte insulin-stimulated glucose uptake, observed both in vivo in mice and in vitro in human adipocytes, led to cell autonomous induction of de novo lipogenesis, which may contribute to improved insulin sensitivity when WAT lipolysis is decreased. The relevance of these observations was established in humans. Investigation of several cohorts of individuals with a wide range of BMI showed a positive association between fat cell lipolytic rate and indexes of insulin resistance and a negative association between lipolysis and expression of genes involved in fatty acid synthesis including the lipogenic transcription factor ChREBP (carbohydrate responsive element-binding protein). Moreover, chronic treatment of obese individuals with an antilipolytic drug resulted in up-regulation of WAT de novo lipogenesis gene expression.

## Results

### Association between WAT Lipolysis and Insulin Sensitivity in Humans

**Figure 1:**
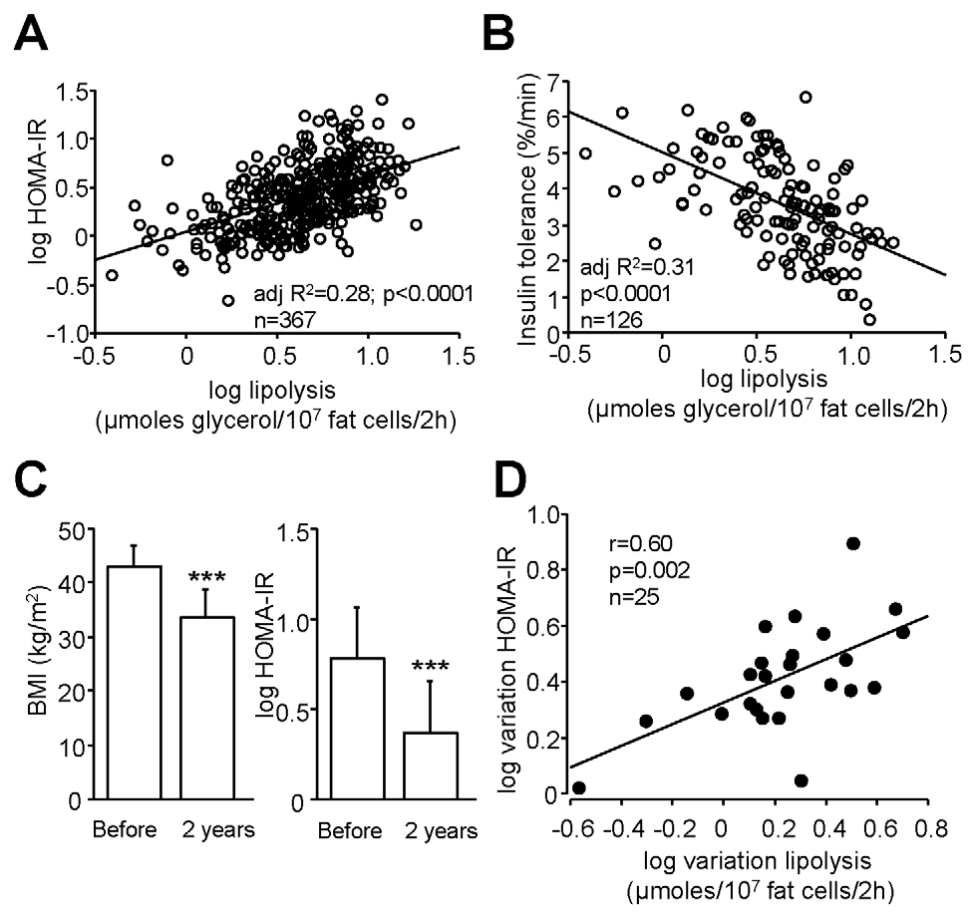
Relationship between WAT lipolytic capacities and insulin sensitivity in human subjects. (A) Simple linear regression between lipolysis and HOMA-IR (*n* = 367). (B) Simple linear regression between lipolysis and insulin tolerance (*n* = 126). (C) BMI and HOMA-IR before and 2 y after bariatric surgery (*n* = 25). (D) Correlation between variations in lipolysis and changes in HOMA-IR following bariatric surgery (*n* = 25).

To investigate the relationship between WAT lipolysis and insulin sensitivity in humans, we first studied a large cohort of subjects presenting a wide range of BMI. A positive association was found in 367 individuals between spontaneous glycerol release measured ex vivo on WAT explants obtained after an overnight fast and the homeostasis model of indirect assessment of insulin resistance (HOMA-IR) (Figure 1A). Variation in lipolysis explained 28% of the variance in HOMA-IR. When adjusted for age, gender, and BMI, 8% of the variance in HOMA-IR remained explained by lipolysis (*p*<0.0001). Dividing the cohort according to WHO criteria for normal weight, overweight, and obesity (i.e., 18.5<BMI<25, 25<BMI<30, 30<BMI<35, 35<BMI<40, BMI>40 kg/m^2^), a correlation coefficient of 0.3 was found for each range of BMI (*p*<0.01), indicating that the relationship between high lipolysis and insulin resistance exists across the spectrum of fat mass. We then assessed insulin tolerance in 126 subjects, after an intravenous bolus injection of insulin. A negative association was found between insulin tolerance and lipolysis (Figure 1B). To gain further insight, this relationship was investigated in 25 morbidly obese individuals who underwent bariatric surgery (Figure 1C). WAT lipolysis and HOMA-IR were measured before and 2 years after bariatric surgery. A correlation was found between the change in lipolysis and the change in HOMA-IR (Figure 1D). The higher the decrease in lipolysis, the stronger the improvement of insulin resistance. Altogether, these data suggest, in both cross-sectional and longitudinal studies, that, in humans, WAT lipolytic capacity may contribute to the control of insulin sensitivity.

### HSL Haploinsufficient Mice Show Reduced Lipolytic Capacities without Alteration of Fat Mass and Adipose Tissue Inflammation

**Figure 2:**
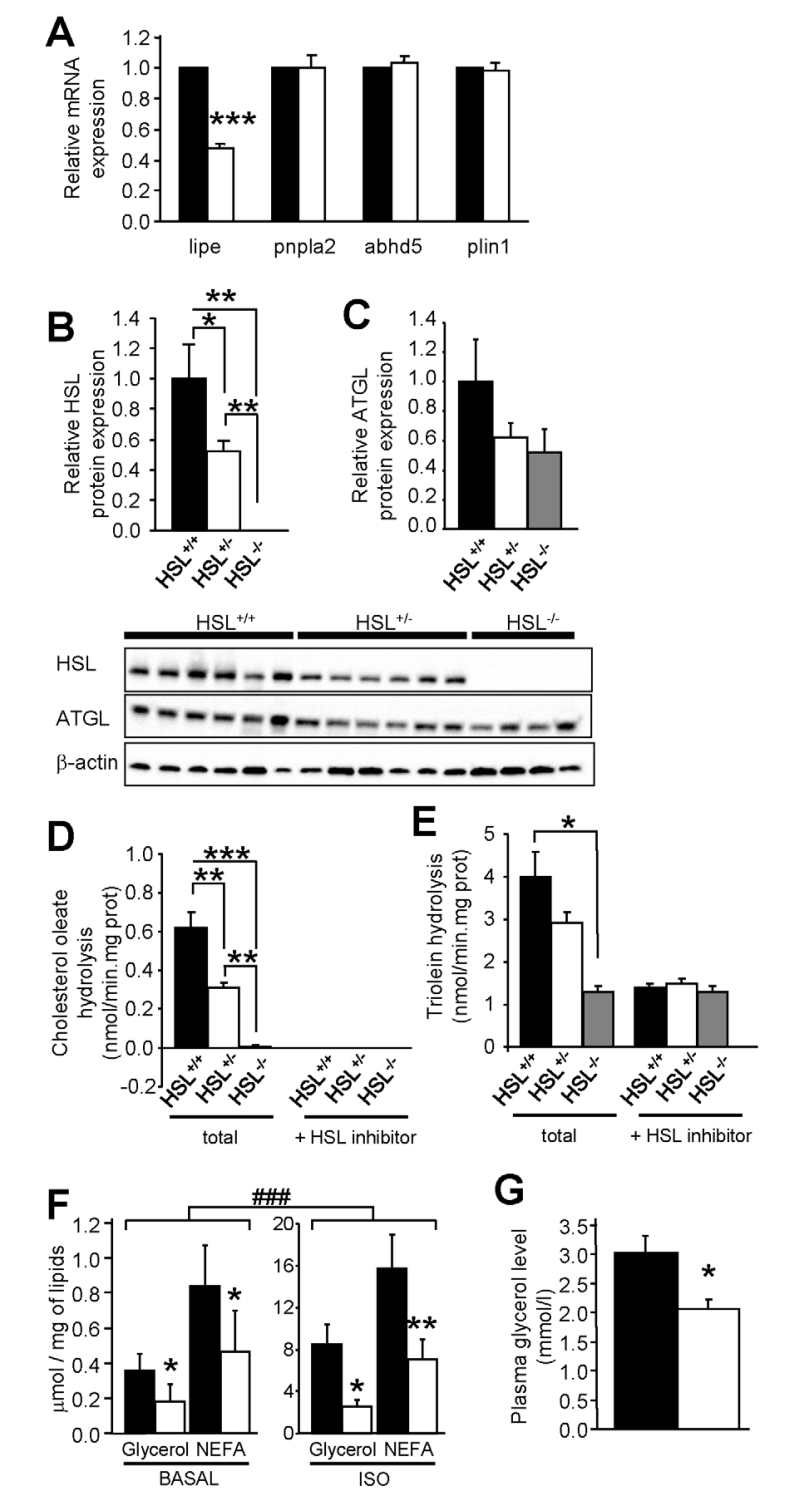
WAT lipases and lipolysis in HFD-fed WT, HSL^+/−^, and HSL^−/−^ mice. (A) mRNA expression of HSL (lipe), ATGL (pnpla2), CGI-58 (abhd5), and plin1 in epididymal WAT. (B–C) Western blot analysis of HSL (B) and ATGL (C) protein expression. (D–E) In vitro hydrolase activities against cholesterol ester (D) and TG (E) analogs in the absence and presence of HSL specific inhibitor. (F) In vitro basal and isoproterenol-stimulated (ISO) lipolysis in isolated adipocytes. (G) In vivo lipolysis expressed as fold increase over saline. Plasma glycerol levels were measured 15 min after saline or isoproterenol injection. Values are means ± SEM. WT mice (black bars) (*n* = 6–8); HSL^+/−^ mice (white bars) (*n* = 6–9); HSL^−/−^ mice (gray bars) (*n* = 4). * *p*<0.05, ** *p*<0.01, *** *p*<0.001 versus WT mice; ^###^ *p*<0.001 versus basal condition.

In search for an animal model with diminished WAT lipolysis and no marked alterations of WAT development, HSL^+/−^ mice were generated by mating WT and HSL^−/−^ mice. HSL (lipe) mRNA expression was 50% lower in epididymal WAT of HSL^+/−^ mice compared to WT mice fed chow and HFD (Figure S1A and Figure 2A, respectively). A similar difference between genotypes was obtained in subcutaneous WAT (unpublished data). mRNA expression of ATGL (pnpla2), its co-activator CGI-58 (abhd5), and perilipin 1 (plin1) was similar in WT and HSL^+/−^ mice, suggesting that no compensatory mechanism occurred as a result of the reduction in HSL expression (Figure 2A and Figure S1A). HSL protein expression was also reduced by 50% in HSL^+/−^ compared to WT mice and undetectable in HSL^−/−^ mice (Figure 2B), whereas ATGL protein expression was not significantly altered in HSL^+/−^ mice (Figure 2C). In vitro total hydrolase activities against cholesterol ester (Figure 2D and Figure S1B) and TG (Figure 2E and Figure S1C) were reduced in WAT of HSL^+/−^ mice, indicating that reduced expression of HSL had the expected impact on cognate enzymatic activities. HSL-specific TG hydrolase activity (expressed as total activity minus activity in the presence of the HSL inhibitor) was significantly decreased in HSL^+/−^ compared to WT mouse WAT (1.45±0.11 versus 2.61±0.52 nmol/min.mg prot, *p*<0.05, respectively). ATGL activity determined in the presence of the HSL inhibitor was not affected (Figure 2E). In HFD-fed mice, consistent with reduced expression and activity of HSL in WAT, adipocytes had reduced in vitro lipolytic response to isoproterenol stimulation (Figure 2F). In addition, in vivo β-adrenergic-stimulated lipolysis was also significantly reduced in HSL^+/−^ mice (Figure 2G). Therefore, decreased expression of HSL alters lipolytic function at a cellular as well as at a organism level.

**Figure 3:**
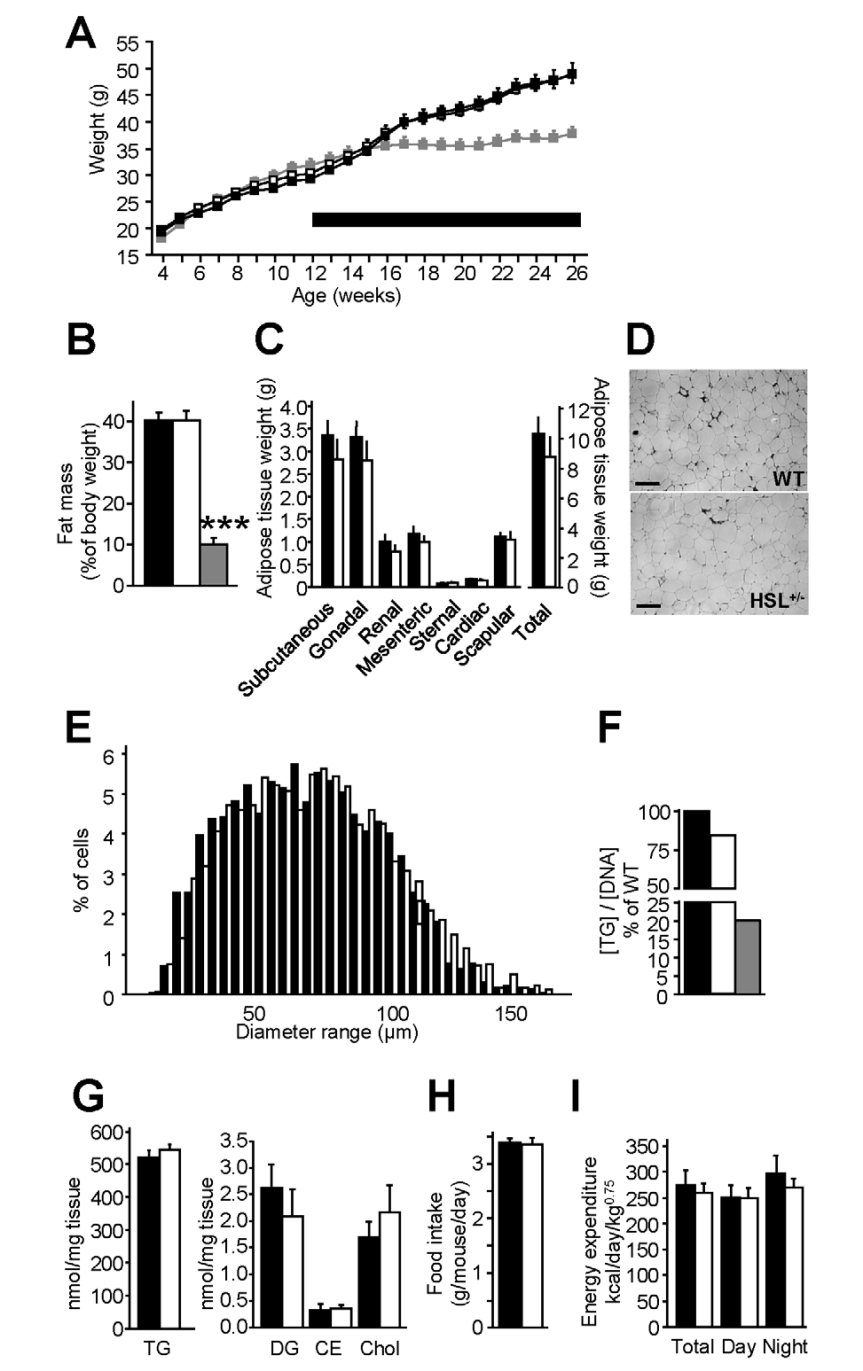
Energy balance of HFD-fed HSL^+/−^ and WT mice. Mice were first provided *ad libitum* access to a standard diet for 8 wk after weaning. Then they received a HFD shown as a thick line for 12 to 16 wk. (A) Body weight curves. (B) Fat mass. (C) Fat pad weight. (D) WAT morphology. Scale bar, 100 µm. (E) Frequency of adipocyte diameter observed on histological preparation of WAT. (F) In vitro adipogenesis of progenitor cells from the subcutaneous WAT stromavascular fraction. Values show results of WAT pools from 2–3 mice. (G) WAT neutral lipids. CE, cholesterol ester; chol, free cholesterol; DG, diacylglycerol; TG, triacylglycerol. (H) Food intake. (I) Energy expenditure assessed by indirect calorimetry. Values are means ± SEM. WT mice (black bars) (*n* = 5–10), HSL^+/−^ mice (white bars) (*n* = 5–10), and HSL^−/−^ mice (gray bars) (*n* = 4). *** *p*<0.001 versus WT mice.

On chow diet, the evolution of body weight (Figure S1D) and fat mass (Figure S1E) were similar in WT and HSL^+/−^ mice. The consequences of diminished HSL function were then investigated in mice fed HFD. As previously reported, HSL^−/−^ mice were paradoxically resistant to diet-induced obesity [13]. WT and HSL^+/−^ mice gained weight at a comparable rate and both became obese (Figure 3A). After 12 weeks of HFD, WT and HSL^+/−^ presented similar fat mass, while fat mass of HSL^−/−^ mice was markedly lower (Figure 3B). Fat pad weights were similar between WT and HSL^+/−^ mice (Figure 3C). Accordingly, plasma levels of leptin were identical in the two genotypes (Table 1). No difference in fat cell morphology was observed by histochemistry (Figure 3D). Mean adipocyte diameter was not modified in HSL^+/−^ mice compared to WT mice (Figure 3E). Differentiation of WAT-derived progenitor cells into adipocytes was similar in WT and HSL^+/−^ mice but markedly reduced in HSL^−/−^ mice (Figure 3F). Profound alterations in WAT gene expression involving PPARγ targets, FA synthesis and esterification, and retinoid and oxidative metabolisms have been reported in HSL null mice [13]–[16]. No alteration was observed in HSL^+/−^ mice (Figure S2). There was no difference in WAT TG, DG, cholesterol ester, or free cholesterol contents between HSL^+/−^ and WT mice (Figure 3G). The assessment of energy balance showed similar food intake and energy expenditure in HSL^+/−^ and WT mice (Figure 3H and Figure 3I). Therefore, the data show that decreased expression of HSL, unlike complete lack of the enzyme, neither influences adipocyte differentiation and fat mass nor alters energy imbalance during diet-induced obesity.

**Table 1:** Fasting plasma parameters in 12-wk wild type (WT) and HSL^+/−^ HFD-fed mice and in 12-wk wild type HFD-fed mice treated with vehicle or HSL specific inhibitor (HSLi) for 7 consecutive days.

	WT	HSL^+/−^	*p*	Vehicle	HSLi	*p*
Glucose (g/l)	1.54±0.11	1.46±0.07	*ns*	1.22±0.05	1.05±0.07	*ns*
Insulin (µg/l)	1.43±0.17	1.18±0.22	*ns*	1.46±0.39	0.88±0.43	*ns*
QUICKI	0.270±0.007	0.280±0.007	*ns*	0.286±0.011	0.376±0.039	0.05
NEFA (mmol/l)	1.02±0.02	0.98±0.10	*ns*	0.88±0.04	0.81±0.05	*ns*
Glycerol (mmol/l)	0.87±0.09	0.88±0.08	ns	0.55±0.06	0.34±0.07	*ns*
Triacylglycerol (mmol/l)	1.43±0.09	1.26±0.10	*ns*	1.63±0.11	1.54±0.10	*ns*
Total cholesterol (mmol/l)	3.07±0.25	3.02±0.24	*ns*	3.33±0.09	3.51±0.09	*ns*
Adiponectin (µg/ml)	5.62±0.20	5.75±0.20	*ns*	6.86±0.53	6.06±0.47	*ns*
Leptin (ng/ml)	28.0±0.9	28.3±0.9	*ns*	17.9±2.5	14.7±2.1	*ns*
RBP4 (µg/ml)	18.0±0.7	18.1±0.6	*ns*	15.2±0.7	15.4±1.0	*ns*

Values (*n* = 6–17) are means ± SEM. NEFA, nonesterified fatty acid; QUICKI, quantitative insulin sensitivity check index; RBP4, retinol binding protein 4.

**Figure 4:**
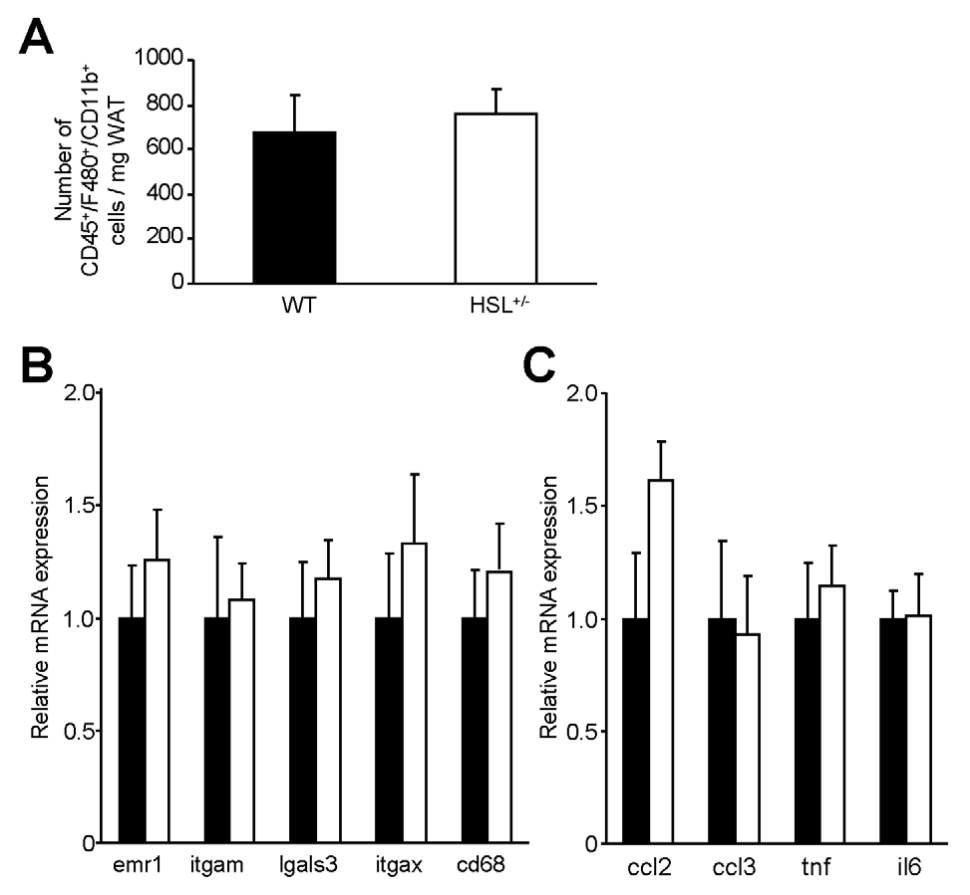
WAT inflammation in 12-wk HFD-fed HSL^+/−^ and WT mice. (A) Macrophage (CD45/F480/CD11b triple positive cells) number per milligram of WAT assessed by flow cytometry. (B) mRNA expression of WAT macrophage surface markers. (C) mRNA expression of WAT inflammatory markers. Values are means ± SEM. WT mice (▪) (*n* = 7) and HSL^+/**−**^ mice (□) (*n* = 8).

Since FA released from adipocytes may modulate WAT inflammation [8],[17], we investigated WAT macrophages and inflammatory molecules. The number of macrophages in the stroma-vascular fraction of WAT did not differ between HSL^+/−^ and WT mice fed a HFD (Figure 4A). Accordingly, mRNA levels of macrophage markers were not different (Figure 4B). Gene expression of inflammatory markers was similar in WAT from HSL^+/−^ and WT mice (Figure 4C). Therefore, the decreased lipolytic capacity of HSL^+/−^ mice does not induce a decrease in WAT inflammation.

### Modification of Fatty Acid Fluxes in HSL Haploinsufficient Mice

**Figure 5:**
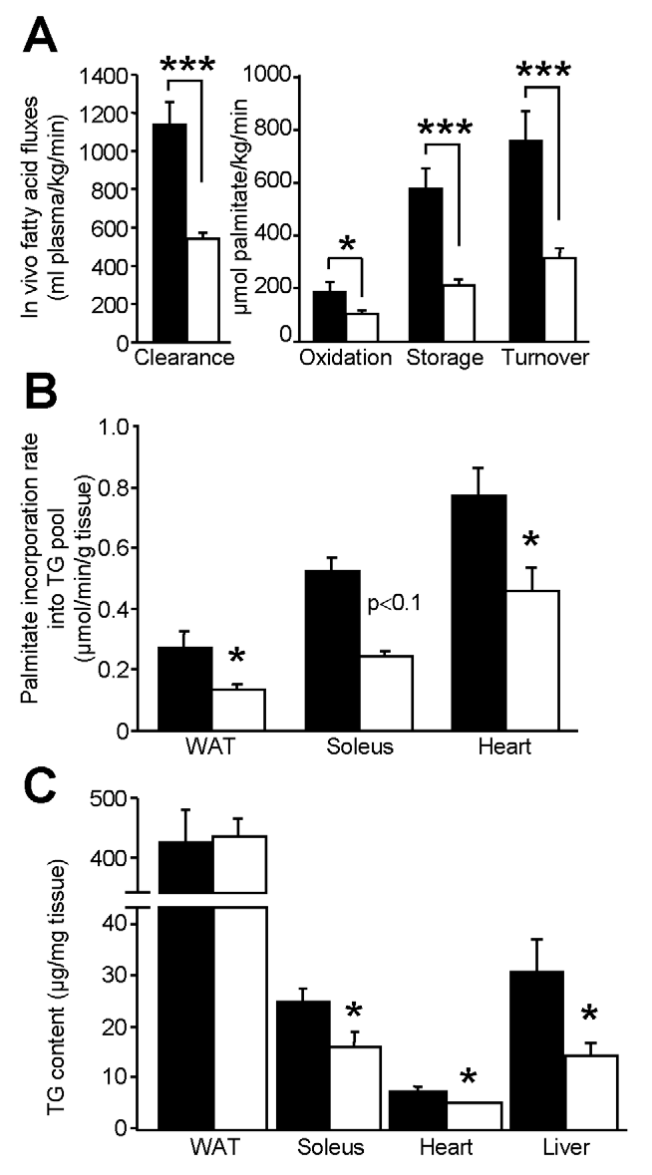
In vivo fatty acid fluxes in HFD-fed HSL^+/−^ and WT mice. (A) Plasma parameters of fatty acid fluxes. (B) Rate of radiolabelled fatty acid incorporation in the TG pool of tissues. (C) Total TG content in tissues. Values are means ± SEM. WT mice (▪) (*n* = 5) and HSL^+/**−**^ mice (□) (*n* = 6). * *p*<0.05, *** *p*<0.001 versus WT mice.

The decreased expression of HSL did not affect fasting plasma parameters (Table 1). NEFA, glycerol, TG, and total cholesterol levels were similar in both genotypes. As no noticeable alteration of metabolic parameters was observed in steady-state measurements, we determined FA fluxes in HFD-fed mice by stable perfusion of radiolabelled palmitate. Global tracer clearance that represents exit of the radioactive tracer from the blood compartment (FA disappearance) is an index of the combined ability of the tissues to take up FAs [18]. Clearance was markedly decreased in HSL^+/−^ mice, indicating that partial HSL depletion has reduced peripheral FA uptake (Figure 5A, left panel). The data suggest that the decreased rate of appearance of FAs in the blood due to decreased WAT lipolysis is, in a steady-state situation with constant plasma NEFA levels, matched by a similar rate of disappearance of FAs (i.e., decreased FA clearance). Global FA oxidation represented by radioactive water measured in plasma was reduced (Figure 5A, right panel). Total radioactive FA storage, deduced from these measurements, was markedly decreased in HSL^+/−^ mice (Figure 5A, right panel). Global FA turnover estimated through the evolution of plasma radioactive palmitate isotopic dilution (which is influenced by the clearance and dilution by cold and radioactive FA released from WAT in the fasted state) was in turn reduced in HSL^+/−^ mice compared to WT mice (Figure 5A, right panel). Therefore, since the mice have been studied in steady-state condition, this suggests that the production rate (WAT lipolysis in accordance with direct measurements shown on Figure 2F, Figure 2G, and liver TG production) was lower as well. Tissue-specific radioactive FA incorporation into the TG pool was evaluated and showed reduction in WAT, heart, and *soleus* muscle of HSL^+/−^ mice (Figure 5B), further supporting the presumed decrease in peripheral FA uptake. Total TG content was not affected in WAT of HSL^+/−^ mice, whereas it was decreased in *soleus* muscle, heart, and liver (Figure 5C). Hence, the decreased lipolytic capacity in WAT induced by partial HSL deficiency provokes a diminution in FA uptake and storage in peripheral tissues. These changes take place without influencing total WAT mass.

### Genetic and Pharmacological Inhibition of HSL Improves Insulin Tolerance and Glucose Metabolism

**Figure 6:**
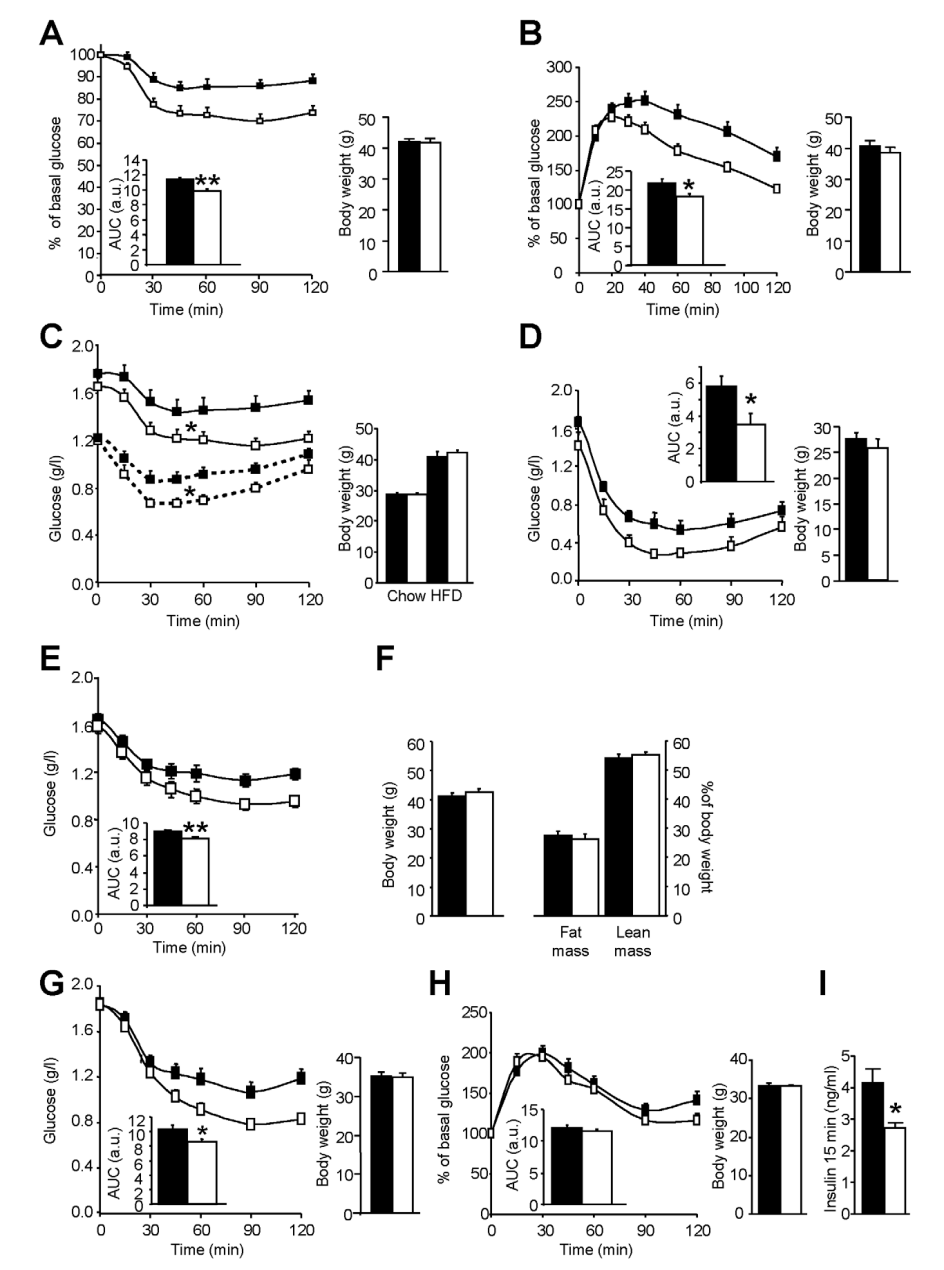
Insulin and glucose tolerance in mice with diminished HSL activity. (A) Insulin tolerance test performed on wild type and HSL^+/−^ mice fed a HFD for 12 wk. (B) Glucose tolerance test performed on wild type and HSL^+/−^ mice fed a HFD for 12 wk. (C) Insulin tolerance test performed on the same animals fed first a chow diet for 6 wk (dotted curve) and then a HFD for 6 wk (solid curve). (D) Insulin tolerance test performed on wild type and HSL^+/−^ mice fed a diet enriched in fructose for 45 wk after weaning. (E–F) Insulin tolerance test (E) and body weight and body composition (F) in 12-wk HFD-fed B6D2 mice treated with a specific HSL inhibitor for 7 d. (G) Insulin tolerance test in 7-wk HFD-C3H/HeJ mice treated with a specific HSL inhibitor for 7 d. (H–I) Glucose tolerance test (H) and plasma insulin at 15 min (I) in LepR^db^/LepR^db^ mice treated with a specific HSL inhibitor for 13 d. AUC, area-under-curve expressed as arbitrary units (a.u.). Body weights at the time of the tolerance tests are shown. Values are means ± SEM. WT mice (▪) (*n* = 6–10) and HSL^+/−^ mice (□) (*n* = 8–10). B6D2 (▪) and HSL inhibitor-treated B6D2 (□) mice (*n* = 10). C3H/HeJ (▪) and HSL inhibitor-treated C3H/HeJ (□) mice (*n* = 8–10). Vehicle- (▪) and HSL inhibitor-treated LepR^db^/LepR^db^ (□) mice (*n* = 12). * *p*<0.05, ** *p*<0.01 versus control mice.

As a negative association was observed in humans between WAT lipolysis and insulin sensitivity (Figure 1), we next investigated peripheral insulin tolerance in HSL^+/−^ mice. Insulin and glucose tolerance tests performed repeatedly on HFD-fed mice revealed that partial HSL depletion improved insulin and glucose tolerance in vivo with no difference in body weight between genotypes (Figure 6A and Figure 6B). However, a gain in peripheral insulin sensitivity could not be observed during hyperinsulinemic euglycemic clamp at 6 mU kg^−1^ min^−1^ of insulin (unpublished data). During clamp studies, insulin is infused at a constant rate to reach a steady state in which WAT lipolysis, which is highly sensitive to the antilipolytic action of the hormone, is strongly suppressed. Indeed, plasma NEFA levels measured during the clamp represented less than one third of fasting levels and were identical in HSL^+/−^ and WT mice (unpublished data). This chronic suppression may explain the lack of differences between genotypes during the clamp studies. Time course insulin tolerance tests first performed on animals fed a chow diet and then challenged after 6 wk of HFD confirmed the protective effect of HSL haploinsufficiency (Figure 6C) and indicated that the relationship between high lipolysis and insulin resistance exists across the spectrum of fat mass in agreement with human data when various ranges of body mass index are considered as reported above. To confirm that HSL haploinsufficiency protects against the development of insulin resistance, WT and HSL^+/−^ mice were fed a high fructose diet known to appreciably alter insulin sensitivity (Figure S3). Insulin tolerance tests performed after 45 wk of diet showed that HSL^+/−^ mice remained more insulin tolerant than WT mice (Figure 6D), supporting the data obtained previously with the fat-enriched diet.

In a therapeutic perspective, we tested the effect of pharmacological inhibition of HSL on insulin tolerance. Twelve-week HFD-fed mice were treated *per os* with a specific HSL inhibitor or vehicle for 7 d [11],[19]. HSL inhibitor-treated animals showed an improvement of insulin sensitivity as shown by QUICKI (Table 1) and of insulin tolerance (Figure 6E) compared to vehicle-treated mice without an effect on body weight and fat mass (Figure 6F). Similar data were obtained on another genetic background (Figure 6G). Treatment with the HSL inhibitor was also performed for 2 wk in 6-wk-old Lepr^db^/Lepr^db^ mice that carry homozygous mutation of the leptin receptor and are genetically prone to obesity and diabetes. The treatment induced no change in body weight. Glycemia was comparable between HSL inhibitor- and vehicle-treated animals at different time points during glucose tolerance test (Figure 6H). However, plasma insulin level at 15 min was lower in mice treated with the HSL inhibitor, indicating a better control of glycemia by insulin when HSL is inhibited (Figure 6I). Similarly to partial genetic HSL depletion, pharmacological HSL inhibition protects mice from insulin and glucose intolerance.

### Molecular Mechanisms Underlying Improvement of Insulin Sensitivity in Mice with Partial Inhibition of HSL

**Figure 7:**
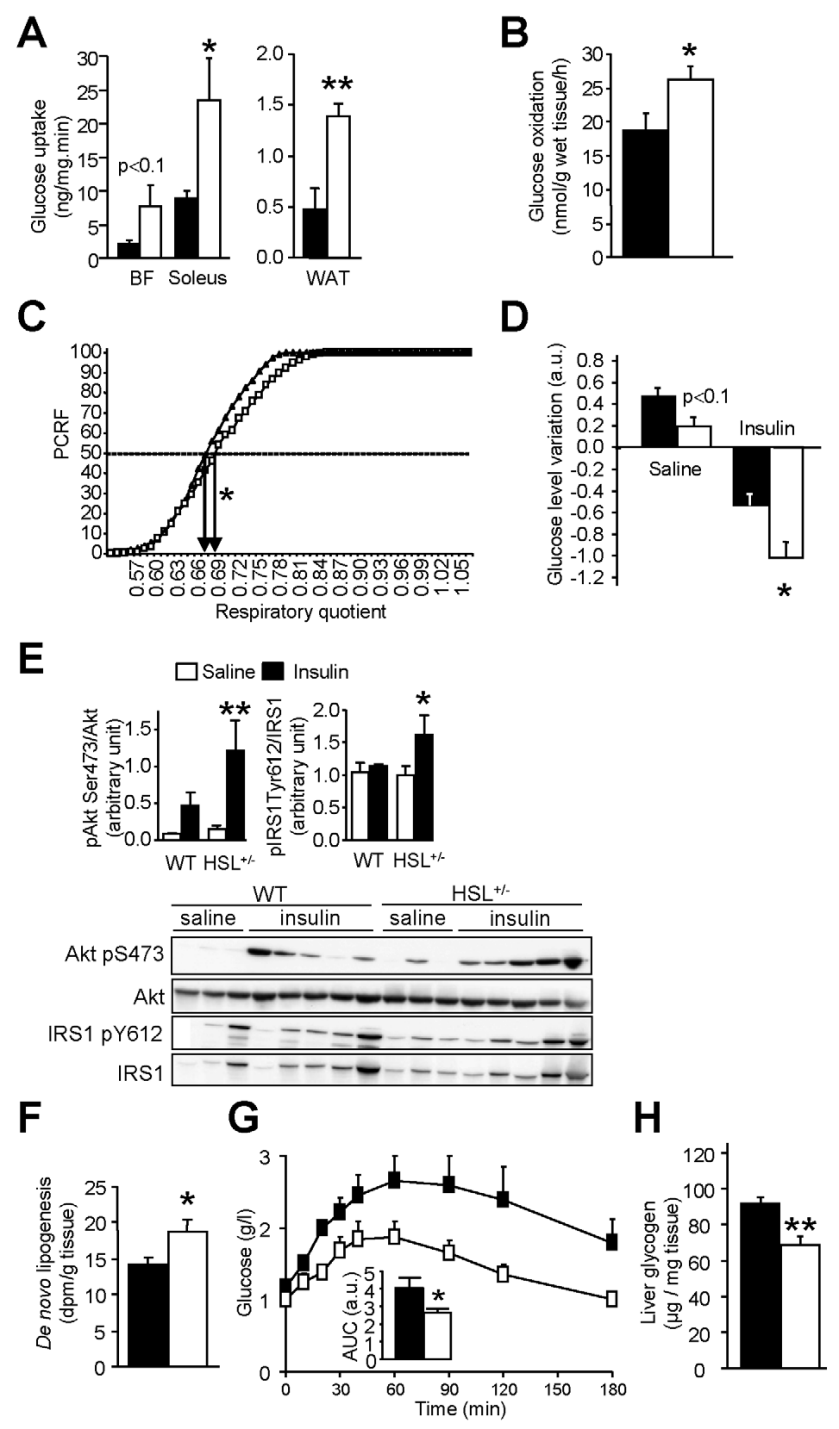
Glucose metabolism and insulin sensitivity in mice with reduced HSL activity. (A) In vivo 2-deoxy-D-[^3^H] glucose uptake under stimulation by insulin in skeletal muscle (*biceps femoris*—BF—and *soleus*) and WAT. (B) Ex vivo glucose oxidation in *soleus* muscle. (C) Respiratory quotient assessed by indirect calorimetry and expressed as percentage of cumulative relative frequencies (PCRF). EC_50_ are represented by arrows. (D) In vivo insulin bolus. Variation in plasma glucose 15 min after injection of saline or insulin (a.u., arbitrary unit). (E) Effect of insulin bolus in vivo on hepatic insulin signalling. IRS1, insulin receptor substrate 1; Akt, protein kinase B. (F) Hepatic de novo lipogenesis. Measurement of radiolabelled glucose incorporation in lipid fraction of liver after insulin stimulation. (G) Pyruvate tolerance test. (H) Liver glycogen content assessed in mice starved for 24 h and then refed for 18 h. Values are means ± SEM. WT mice (▪ or ▴) and HSL^+/−^ mice (□) mice (*n* = 4–10 in each group). * *p*<0.05, ** *p*<0.01 versus WT mice.

The improvement of insulin sensitivity in HFD-fed HSL^+/−^ and HSL inhibitor-treated mice could result from changes in levels of adipokines with action on insulin signalling. Adiponectin plasma levels were not different from control animals in mice with partial genetic or pharmacologic inhibition of HSL (Table 1). Similarly, plasma concentrations of retinol binding protein 4 (RBP4), an adipokine involved in the development of insulin resistance [20], were not modified in HSL^+/−^ and HSL inhibitor-treated mice compared to control mice. To shed light on the origin of the global improvement in insulin tolerance in HSL^+/−^ mice, in vivo insulin-stimulated glucose uptake was determined in various tissues. Glucose uptake was increased in *soleus* (oxidative) muscle and showed a tendency to increase in *biceps femoris* (glycolytic) muscle (Figure 7A). An increase in glucose uptake was also observed in WAT. Furthermore, glucose oxidation measured ex vivo in *soleus* muscle was increased (Figure 7B). There were no differences in DG and glycogen contents between HSL^+/−^ and WT skeletal muscle (unpublished data). Interestingly, respiratory quotient was increased in HSL^+/−^ mice showing a shift from FA to glucose as energy substrate (Figure 7C). To determine whether liver was involved in the improvement of glucose and insulin tolerance in HSL^+/−^ mice, we administered a bolus of insulin and measured the effects on the hepatic insulin signalling pathway. In agreement with an improved insulin tolerance, the decrease of glycemia was greater in HSL^+/−^ mice than in WT mice (Figure 7D). In response to exogenous insulin administration, levels of phosphorylated insulin receptor substrate 1 and Akt were increased in the liver of HSL^+/−^ mice supporting an improvement of insulin signalling (Figure 7E). HSL^+/−^ mice infused with radiolabelled glucose showed a rise in glucose carbon incorporation into hepatic lipids—that is, de novo lipogenesis—compared to WT mice (Figure 7F). Pyruvate tolerance test revealed that gluconeogenesis was reduced in HSL^+/−^ mice (Figure 7G). The reduced hepatic glucose production was associated with decreased glucose storage as demonstrated by reduced glycogen content in HSL^+/−^ mice (Figure 7H). Liver DG content was unchanged in HSL^+/−^ mice (unpublished data). As changes in the capacity of the pancreas to produce insulin could modify glucose tolerance, we then investigated pancreatic function in vivo and in vitro. Neither arginine tolerance test performed on 12-wk HFD-fed animals (Figure S4A) nor in vitro glucose-stimulated insulin secretion of isolated pancreatic islets (Figure S4B) revealed changes in insulin secretion in HSL^+/−^ compared to WT mice, arguing against a direct effect of HSL haploinsufficiency on the capacity of pancreatic β cells to secrete insulin. Altogether, adaptations in WAT, skeletal muscle, and liver explain the global improvement in glucose and insulin tolerance observed in mice with HSL haploinsufficiency.

### HSL Gene Silencing in Vitro and Partial Inhibition of Lipolysis In Vivo Affects Nutrient Partitioning and Promotes De Novo Lipogenesis in Human Adipocytes

**Figure 8:**
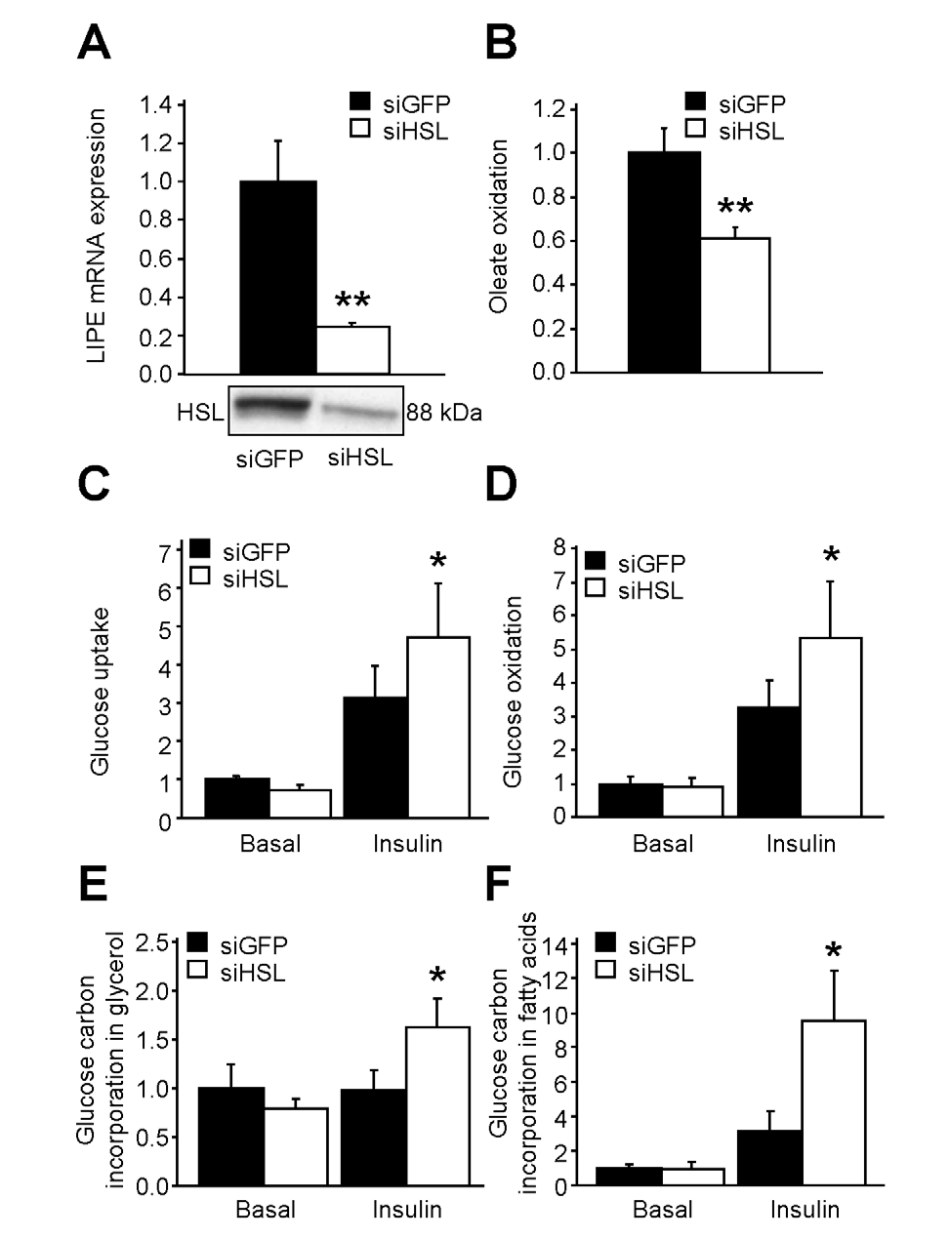
Fate of glucose and fatty acid in hMADS adipocytes. (A) HSL (LIPE) expression as mRNA (upper panel) and protein (lower panel). (B) Fatty acid oxidation. (C) Basal and insulin-stimulated glucose uptake. (D) Basal and insulin-stimulated glucose oxidation. (E–F) Basal and insulin-stimulated glucose carbon incorporation into glycerol (E) or fatty acid (F) of neutral lipids. hMADS adipocytes were transfected either with a siRNA against GFP (control; siGFP) or HSL (siHSL). Data are fold induction of siGFP values (means ± SEM). siGFP adipocytes (▪) and siHSL adipocytes (□) (*n* = 5–10). * *p*<0.05, ** *p*<0.01 versus siGFP.

**Figure 9:**
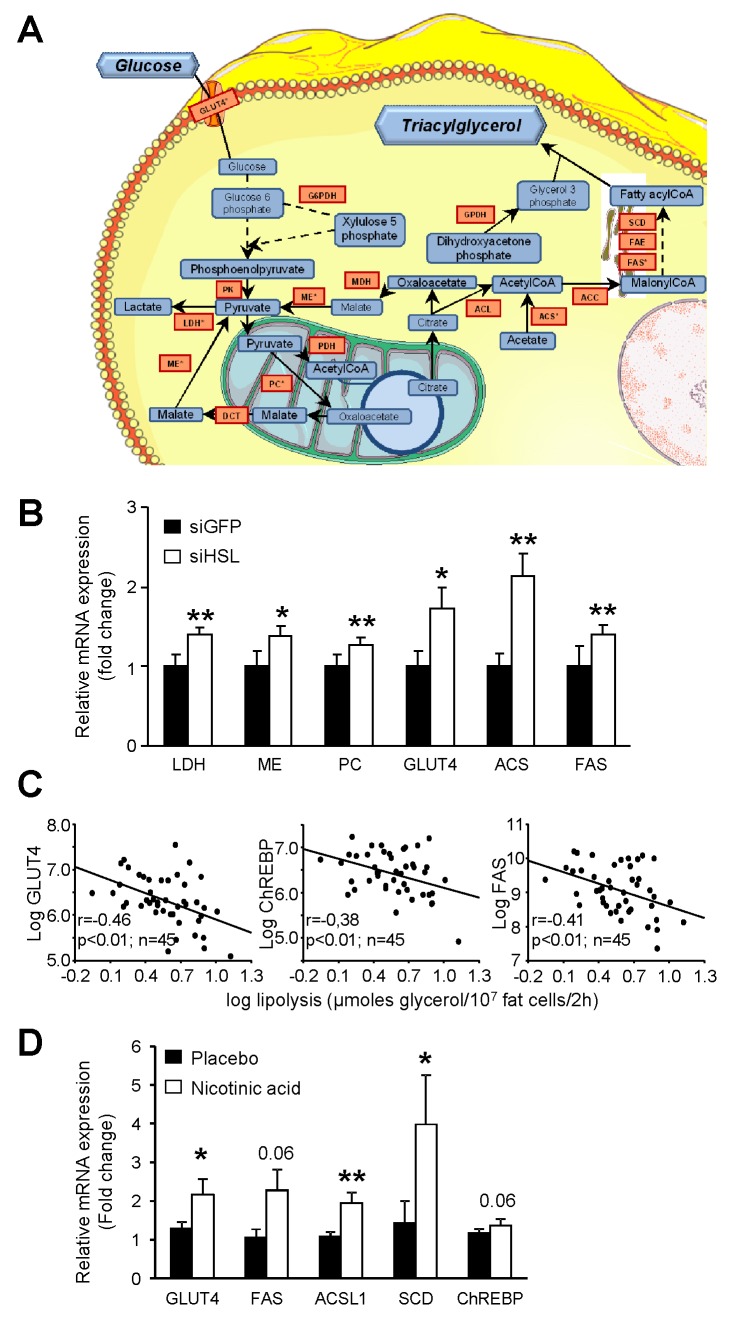
Relationship between WAT lipolysis and de novo lipogenesis. (A) Up-regulation of glucose transporter 4 and de novo lipogenesis-related pathways. Induction of gene expression (red boxes) in hMADS adipocytes with HSL gene silencing were determined by DNA microarray analysis and validated by reverse transcription-quantitative PCR (indicated by *). (B) Up-regulation of glucose transporter 4 and de novo lipogenesis-related pathway gene expression in hMADS adipocytes. mRNA levels were determined by reverse transcription-quantitative PCR (*n* = 9–12). ** *p*<0.01 versus siGFP. (C) Correlations between glucose transporter 4, carbohydrate responsive element-binding protein, and fatty acid synthase mRNA levels and, lipolysis in human WAT (*n* = 45). (D) Up-regulation of glucose transporter 4 and de novo lipogenesis-related pathway gene expression in adipocytes from obese individuals treated with placebo or nicotinic acid (*n* = 12 per group). * *p*<0.05, ** *p*<0.01 versus before treatment. ACC, acetylCoA carboxylase; ACL, ATP citrate lyase; ACS, acetyl-CoA synthase; ChREBP, carbohydrate responsive element-binding protein; DCT, dicarboxylate transporter; FAE, fatty acid elongase; FAS, fatty acid synthase; GPDH, glycerol-3-phosphate dehydrogenase; GLUT4, glucose transporter 4; G6PDH, glucose-6-phosphate dehydrogenase; LDH, lactate dehydrogenase; MDH, malate dehydrogenase; ME, malic enzyme; PC, pyruvate carboxylase; PDH, pyruvate dehydrogenase; PK, pyruvate kinase; SCD, stearoylCoA desaturase.

In order to determine whether modifications of fat cell metabolism observed in vivo in HSL^+/−^ mice were cell autonomous, expression of HSL was knocked down in human hMADS adipocytes. Adipocytes transfected by HSL siRNA showed a 70% reduction in LIPE mRNA expression compared to GFP siRNA transfected cells (Figure 8A, upper panel). HSL protein expression was also reduced in HSL-silenced adipocytes (Figure 8A, lower panel). As observed at the whole body level in HSL^+/−^ mice (Figure 5A), HSL gene silencing resulted in a decrease of FA oxidation in human adipocytes (Figure 8B). HSL gene silencing also led to an increase in insulin-stimulated glucose uptake (Figure 8C) as shown in vivo in HSL^+/−^ mouse WAT (Figure 7A), accompanied by a rise in insulin-stimulated glucose oxidation (Figure 8D). The enhanced influx of glucose was associated with an increase in glyceroneogenesis and de novo lipogenesis in adipocytes knocked down for HSL (Figure 8E and Figure 8F). Expression of the glucose transporter GLUT4 and of glycolytic and fatty acid synthesis genes showed coordinated up-regulation in HSL-silenced adipocytes (Figure 9A and Figure 9B). RBP4 mRNA (1.4-±0.2-fold siHSL/siGFP, *n* = 9, *p*<0.05) and secreted protein levels (1.2-±1.0-fold siHSL/siGFP, *n* = 11, *p*<0.001) were slightly but significantly increased in hMADS adipocytes transfected with HSL siRNA (Figure S5). This piece of data does not support a role of this adipokine in the improvement of insulin action and glucose metabolism when HSL expression is lowered. As glucose uptake and fatty acid synthesis were upregulated in vitro in human adipocytes with low HSL level, we investigated correlations between lipolysis and expression of key genes of these pathways in vivo in human WAT. Simple regression analysis showed negative associations between GLUT4, fatty acid synthase or the lipogenic transcription factor ChREBP mRNA levels, and WAT lipolytic rates (Figure 9C). The negative associations persisted in multiple linear regression analyses with BMI as the covariate (partial *R* = 0.3, *p*<0.05). No association was found between mRNA level of SREBP1c, another transcription factor controlling expression of FA synthesis genes, and lipolysis (*p* = 0.96). As further proof supporting the link between human fat cell lipolysis and fat cell de novo lipogenesis, 8-wk treatment of obese male individuals with nicotinic acid, which inhibits lipolysis through activation of a fat cell Gi protein-coupled receptor [1], resulted in up-regulation of adipocyte de novo lipogenesis gene expression (Figure 9D).

## Discussion

**Figure 10:**
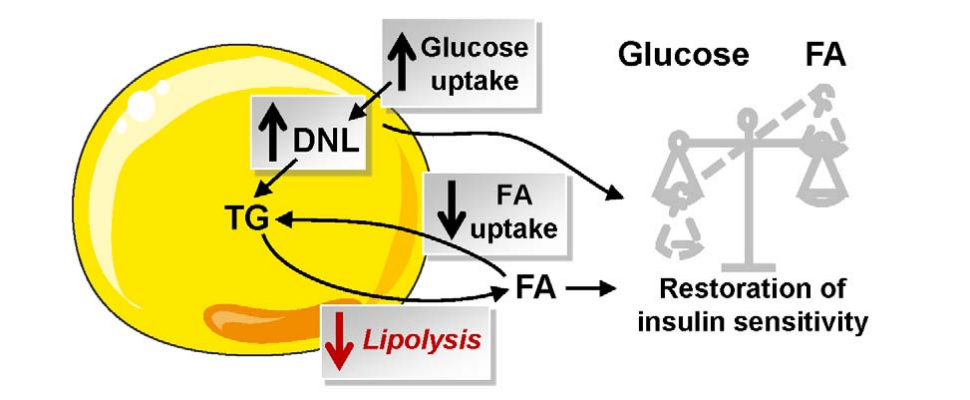
Consequence of partial inhibition of WAT lipolysis on fatty acid and glucose metabolism and insulin sensitivity. Partial inhibition of lipolysis modifies FA flux with a decrease in WAT FA uptake concurring to maintenance of fat mass and an increase in glucose uptake that favours de novo lipogenesis, which may be a key player in the restoration of insulin sensitivity. DNL, de novo lipogenesis; FA, fatty acid; NEFA, nonesterified fatty acid; TG, triacylglycerol.

Insulin resistance is a critical pathogenic process linking obesity to type 2 diabetes and cardiovascular diseases. To date, there is convincing evidence in humans that FAs cause deleterious effects on insulin signalling in peripheral organs [21]. The working models are based on overload of lipids from exogenous sources—that is, dietary FA for HFD or FA produced by lipoprotein lipase-mediated hydrolysis of TG during lipid and heparin infusion [7],[22]. However, little is known on the effect of FAs released by WAT lipolysis on the modulation of fat mass and insulin sensitivity. The physiological significance of lipolysis may be seen in two, not necessarily mutually exclusive, ways. Impaired lipolytic capacity could contribute to the development of obesity through impairment in the mobilization of fat stores. Alternatively, the defect may protect against excessive FA release and ensuing deleterious action of FAs on insulin sensitivity. To address this clinically relevant question, we used a translational approach combining human and animal studies together with cellular investigations. As we previously showed that the level of HSL expression controls lipolytic rate in human fat cells and is altered in obesity [10]–[12], the association between WAT lipolysis, fat mass, and insulin sensitivity was investigated in mice with HSL haploinsufficiency. To address this relationship, lipase total knockout mouse models may not prove suitable. HSL null mice show impaired development of WAT when fed a HFD and marked WAT inflammation in the absence of obesity [13],[23]. Metabolic defects are observed in multiple organs of ATGL global knockout mice, causing a complex modulation of insulin sensitivity [9],[24]–[26]. WAT-specific ablation of ATGL shows markedly altered thermogenesis [27]. Therapeutic relevance was provided by studies on mice treated with a specific HSL inhibitor. Cell-autonomous effects of HSL deficiency were investigated in hMADS adipocytes, a validated model for the study of human fat cell metabolism [2],[28]. The relationship between WAT lipolysis and glucose metabolism was further explored in four independent cohorts of individuals with a wide range of BMI. The results are summarized on Figure 10.

Phenotyping obese mice with reduced HSL activity in WAT first revealed that a partial defect in lipolysis did not modify adiposity and, hence, that chronic inhibition of FA release from WAT did not contribute to the development of obesity. Strikingly, complete HSL deficiency leads to resistance to HFD-induced obesity [13] and impaired adipogenesis (present work). However, the increase in body fat mass, weights of fat depots, and adipocyte size was not compromised in HSL^+/−^ mice fed HFD. It may be hypothesized that the presence of an active allele is sufficient to compensate for the defect in adipogenesis, which has been linked to an impaired production of signalling lipolytic by-products [16],[29]. The identical fat mass in HSL^+/−^ and WT mice was supported by similar food intake, energy expenditure, and leptin levels in the two genotypes. The normal development of WAT in a condition of diminished adipose lipolysis raised questions on the dynamics of lipid fluxes. Using a fluxomics approach [18], we show that HSL^+/−^ mice presented altered global FA turnover, decreased WAT lipolysis being balanced by reduced FA esterification in WAT. Accordingly, we have previously reported a coupling between FA release and re-esterification in vitro in human adipocytes [2]. The decreased FA oxidation observed in human adipocytes with altered lipolytic capacity may result from decreased FA availability. In heart and brown adipose tissue, it has been postulated that FA need to be esterified into TG and then mobilized for being properly oxidized in mitochondria [30],[31]. Moreover, enhanced de novo lipogenesis in adipocytes knocked down for HSL results in an increase of the levels of malonyl-CoA, which inhibits the rate-limiting step in FA oxidation, carnitine palmitoyl transferase 1b [32]. Therefore, partial inhibition of WAT lipolysis results in a modification of FA fluxes in vivo and in fat cells without alteration of fat mass.

Our data in humans show, on a large number of individuals, a strong association between WAT lipolysis and indexes of insulin resistance independently of fat mass. This relation was confirmed in a longitudinal study. Two years after bariatric surgery in morbidly obese individuals, the diminution of lipolytic rate was positively correlated with the improvement of insulin sensitivity. In obese HSL haploinsufficient mice, we observed an improvement of glucose metabolism and insulin tolerance. It happens with a global slowdown of FA turnover and unchanged plasma FA levels in the overnight fasted state, a condition observed in many obese individuals [6]. Results from insulin and glucose tolerance tests were supported by organ-specific adaptations. In the liver, insulin action was improved as revealed by an increase in tyrosine phosphorylation of insulin receptor substrate 1 and serine phosphorylation of Akt. An improvement in hepatic insulin sensitivity has been reported in one model of total HSL deficiency [33]. Hepatic glucose fluxes were modified in HSL haploinsufficient mice. The decrease in glucose production, which could partially result from the reduced glycerol availability, is balanced by a decrease of glucose storage as glycogen. The shift in respiratory quotient could sign an increase in carbohydrate usage and decreased lipid utilisation but could also result from the conversion of carbohydrate to lipid and its subsequent oxidation as this process has the same respiratory quotient as direct oxidation of carbohydrates. Accordingly, *soleus* muscle glucose oxidation and hepatic de novo lipogenesis were increased in HSL^+/−^ mice. In vivo insulin-stimulated glucose uptake was increased in WAT and skeletal muscles of HFD-fed HSL^+/−^ mice, suggesting an improvement in peripheral insulin sensitivity. It is now well established that lipid-induced insulin resistance in skeletal muscle stems from defects in insulin-stimulated glucose transport [7]. Moreover, a better glucose uptake in fat cells may participate in the amelioration of insulin sensitivity at the whole body level. Indeed, adipocyte-specific invalidation of GLUT4 abolishes insulin-stimulated glucose uptake in WAT and impairs insulin action in liver and skeletal muscle [34]. RBP4 was described as a potential mediator of insulin resistance in these mice [20]. However, in vivo data in mice as well as in vitro data in human adipocytes show that it is unlikely that RBP4 contributes to the improvement of insulin tolerance and glucose metabolism promoted by diminished WAT lipolysis. The rise in insulin-stimulated glucose uptake was also observed in a cell-autonomous manner in human adipocytes with diminished HSL expression. It was accompanied by increased glucose oxidation and glucose carbon incorporation into FA and glycerol—that is, increased de novo lipogenesis and glyceroneogenesis. Glucose uptake controls adipocyte de novo lipogenesis, which has recently appeared as a major determinant of whole body insulin sensitivity [35],[36]. The negative correlations between GLUT4, ChREBP, or fatty acid synthase mRNA levels and WAT lipolytic rate in vivo in humans strongly support that notion. As further proof of concept, chronic inhibition of WAT lipolysis with nicotinic acid resulted in an increase of fat cell de novo lipogenesis gene expression in obese individuals. In HFD-fed obese HSL^+/−^ mice, the concomitant up-regulation of de novo lipogenesis in WAT and liver may play an important part in the favourable metabolic profile [35],[37],[38]. Altogether, the modifications in FA metabolism resulting in enhanced fat cell glucose uptake and de novo lipogenesis are therefore likely to contribute to the improvement of insulin sensitivity.

Increase in WAT macrophage number and expression of immune cell-derived cytokines and chemokines may contribute to obesity-induced insulin resistance [39]. As FAs released from 3T3-L1 adipocytes have been shown to stimulate pro-inflammatory cytokine production by RAW264 macrophages, it could be hypothesized that inflammation would be diminished in WAT from obese HSL^+/−^ mice [8]. However, neither macrophage number nor gene expression of macrophage markers and inflammatory factors were modified, indicating that chronic inhibition of lipolysis, unlike acute stimulation, does not modify the content of macrophages in WAT [17]. In HSL^+/−^ mice, the lack of alteration in WAT inflammation is, however, in line with the lack of change in fat mass.

This work has relevance on therapeutic strategies aimed at preventing the development of obesity-associated insulin resistance. The interest in antilipolytic drugs has been shown with nicotinic acid, which has been used for decades as a lipid-lowering drug. This compound acts through a G-protein-coupled receptor with antilipolytic action in fat cells [1]. However, the receptor is expressed in other cell types than adipocytes, and nicotinic acid shows receptor-independent effects in the liver. The use of the drug has been restricted due to upper-body skin flushing. Moreover, data on insulin sensitivity are conflicting [40]. A search for alternative drugs with an antilipolytic effect has led to synthesis of several series of HSL inhibitors [41]. The compounds are highly selective in part because of the low homology between HSL and known mammalian lipases [42]. We show here that chronic pharmacological inhibition of lipolysis using a selective HSL inhibitor improved insulin action in HFD-fed mice and genetically obese LepR^db^/LepR^db^ mice. From the clinical data presented here, it appears that insulin-resistant individuals with a high lipolytic rate will benefit the most from treatment with antilipolytic molecules such as HSL inhibitors. An additional clinical advantage with HSL inhibition is the lack of effect, at least in mice, on fat mass. As discussed earlier, plasma levels of FA poorly correlate with insulin sensitivity [6]. Our data do not dispute this notion but suggest that it is FA fluxes rather than FA levels that determine insulin sensitivity.

In summary, a decrease in WAT lipolysis results in a slowdown of FA turnover associated with improved insulin tolerance and glucose metabolism and no change in fat mass (Figure 10). Long-term moderate inhibition of WAT lipolysis can therefore be beneficial in the treatment of obesity-related insulin resistance.

## Materials and Methods

### Ethics Statement

Clinical studies were approved by the ethics committee of Karolinska Institute University Hospital and Toulouse University Hospitals. Informed consent was obtained from each participant.

All experimental procedures on mice were performed according to INSERM and Genotoul Anexplo animal core facility guidelines for the care and use of laboratory animals.

### Generation of Transgenic Mice, Treatments, and Phenotypic Analyses

Targeted disruption of the HSL gene and generation of HSL^−/−^ mice have been described elsewhere [43]. Four- to 5-wk-old B6D2 mice were fed chow diet (10% kCal fat, D12450B, Research Diets Inc.) for 6 or 28 wk, HFD (45% kCal fat, D12451, Research Diets Inc.) for 12 to 16 wk, or fructose-enriched diet (D11743, Research Diets Inc.) for 45 wk. C3H/HeJ mice were purchased from Jackson Laboratories. They were fed a HFD for 7 wk. For fasting-refeeding experiments, 12-wk HFD-fed WT and HSL^+/−^ male mice were overnight fasted and refed for 18 h before sacrifice. For chronic HSL inhibition, the specific HSL inhibitor synthesized by IDEALP PHARMA was given orally at 70 mg/kg/d [19]. B6D2 and LepR^db^/LepR^db^ mice were, respectively, treated once daily during 7 or 13 consecutive d. Body weight was measured weekly. Food intake was measured daily during 4 d in animal housed individually. Body mass composition was evaluated by quantitative nuclear magnetic resonance system (EchoMRI 3-in-1, Echo Medical Systems). In vivo lipolytic challenge by intraperitoneal (ip) injection of the β-adrenergic agonist isoproterenol (10 mg/kg) on mice fasted for 7 h was performed as previously reported [44].

For ip insulin tolerance tests, an injection of 0.6 U/kg insulin was given to 6-h–fasted mice. For oral glucose tolerance tests, an oral administration of 1.5 g/kg D-Glucose was given to 16-h–fasted mice with, in LepR^db^/LepR^db^ mice, supplemental blood sampling performed at 15 min for insulin measurement. For pyruvate tolerance test, an ip injection of 2 g/kg pyruvate was performed on 16-h–fasted mice. Blood glucose levels were monitored from the tip of the tail vein with a glucometer (Accucheck, Roche). Arginine tolerance test was performed on WT and HSL^+/−^ mice fed a HFD for 12 wk. The 16-h–fasted mice anesthetized with isoflurane were ip injected with 3 g/kg of L-arginine (Sigma). Blood sampling for insulin measurement was performed at the retro-orbital sinus.

For the measurement of in vivo glucose utilization in individual tissues, an intravenous bolus of 50 µCi 2-deoxy-D-[^3^H] glucose (Perkin Elmer) was given during euglycemic hyperinsulinemic clamp (6 mU kg^−1^ min^−1^ of insulin). Disappearance of plasma 2-deoxy-D-[^3^H] glucose and glucose concentrations were determined in 5 µl blood samples from the tail vein. Different tissues were dissected and dissolved in 1 M NaOH during 1 h. 2-deoxy-D-[^3^H] glucose 6-phosphate and 2-deoxy-D-[^3^H] glucose were differentially precipitated by the use of zinc sulfate (0.3 M), barium hydroxide (0.3 M), and perchloric acid solutions (6%).

For the measurement of in vivo hepatic de novo lipogenesis, livers were harvested after an euglycemic hyperinsulinemic clamp (1.5 mU kg^−1^ min^−1^ of insulin) with D-[3-^3^H]glucose (Perkin Elmer) infusion at 15.9 kBq/min. After homogenization in a lysis buffer, lipids were extracted by a Folch method and the organic phase was counted for radioactivity.

For in vivo measurements of phospho-insulin receptor substrate 1 and phospho-Akt, animals fed a HFD for 12 wk were fasted for 6 h before ip injection of 10 U/kg insulin. Liver was harvested 15 min after injection. Liver proteins were solubilised in RIPA buffer containing protease and phosphatase inhibitors. Protein samples were resolved by SDS-PAGE, blotted, and incubated with anti-phospho insulin receptor substrate 1 (Tyr^612^), phospho Akt (Ser^473^), total insulin receptor substrate 1, or total Akt antibodies (Cell Signaling). Equal loading was confirmed using anti-GAPDH protein.

To investigate FA fluxes, a catheter was inserted into the femoral vein in HSL^+/−^ and WT animals under isoflurane anaesthesia. Mice were allowed to recover for 4–5 d before assessing FA fluxes in awake free-moving mice. Mice were then infused in fasting conditions (during 6 h from 08:00) with a tracer solution freshly prepared each day. Infusate was prepared with 50 µM palmitate and [9,10-^3^H] palmitic acid. Infusions were performed at a constant rate of 0.2 µCi/4 µl/min for 2 h. Blood samples (30 µl) were collected from the tip of the tail at −30, 0, 60, 75, 90, and 120 min. Tracer infusion was stopped at 120 min, and additional blood samples were collected at 125, 130, and 140 min. Blood samples were rapidly centrifuged to prepare plasma, which was kept at −80°C until biochemical measurements. Total plasma NEFA and TG were measured with a colorimetric enzymatic method (respectively, NEFA C, Wako, and TG PAP150, Biomerieux). Lipid extraction and separation procedure were performed to determine plasma ^3^H_2_O, ^3^H-NEFA and ^3^H-TG as described by Oakes et al. [18]. During a first step, ^3^H_2_O was separated from lipid phase using an isopropanol-hexane-H_2_SO_4_ 0.5 M mixture. In a second step, TGs were separated from neutral lipids using an alkaline methanol solution. Finally, NEFAs were extracted from neutral lipid phase using an acid hexane solution. Radioactivity was counted in each fraction. Whole body rates of FA clearance, appearance, oxidation, and storage were determined as described by Oakes et al. [18]. At 140 min, the last blood sample was collected from retro-orbital sinus. Mice were euthanized with an intravenous bolus of pentobarbital. Different tissues were collected and weighed before freezing in liquid nitrogen and storage at −80°C for total TG content assessment. A sample of WAT, *soleus* muscle, and heart was homogenized in water. Non-^3^H_2_O products were extracted using an isopropanol-hexane-H_2_SO_4_ 0.5 M mixture, and radioactivity was counted. NEFA incorporation rates into tissue-specific storage products were calculated between 120 and 140 min.

Oxygen (VO_2_) and carbon dioxide (VCO_2_) production was measured using a four-chamber oxylet system (Bioseb). Temperature was maintained at 21±1°C, and the light was on from 07:00 to 19:00. System setting included a flow rate of 0.3 l/min, a sample purge of 5 min, and a measurement period of 5 min every 25 min. Twenty-four h prior to data collection, mice were placed in separate calorimetry chambers (each with a volume of 2.5 l), with free access to food and water. The respiratory quotient was calculated as the ratio of VCO_2_/VO_2_; results were expressed as percent of relative cumulative frequency along the measurement period [45].

For measurements of plasma parameter concentrations, insulin, NEFA, and glycerol were determined by ELISA (Mercodia), an enzymatic colorimetric reaction (NEFA C, Wako), and with the hydrazine buffer method or with a commercial kit (Sigma), respectively. TG and cholesterol were determined with a COBAS-MIRA + analyzer (ABX Diagnostics). Adiponectin, leptin, and RBP4 were measured with commercial ELISA kits (R&D Systems).

### In Vitro Measurements on Mouse Tissues

Overnight fasted mice were euthanized at various weeks of age depending on experiments, blood was collected in EDTA tubes, and various tissues were removed, immediately weighed, frozen in liquid nitrogen, and stored at −80°C.

Epididymal fat samples were fixed in 1% formalin (Sigma), embedded in paraffin, and processed to hematoxylin and eosin staining. Digital images were captured using light microscope coupled to a camera and analyzed using a morphometric programme (Lucia IMAGE, version 4.81; Laboratory Imaging). Adipocyte size was determined on a histological preparation measuring area of at least 200 adipocytes.

In adipogenesis test, the stromavascular fraction (SVF) was isolated from subcutaneous WAT of 12-wk HFD-fed WT, HSL^+/−^, and HSL^−/−^ mice. WAT from 2–3 mice were pooled together. SVF cells were plated at a density of 70,000 cells per well, in a 48-well plate, in 10% FBS-endothelial cell basal medium. Media were replaced at Day 1 and Day 4. At Day 5, the medium was replaced with 2% FBS—endothelial cell basal medium supplemented with 20 nM insulin, 0.2 nM T3, 100 nM cortisol, 0.01 mg/ml transferrin, and then changed every 2 d. At Day 14, lipid accumulation was estimated through Oil Red O staining normalized by total DNA amount measured using PicoGreen (InVitrogen).

For flow cytometry analysis of WAT, SVF cells were obtained by collagenase digestion as previously described [46]. After digestion, the suspension was filtered through 150 µm sieves and centrifuged (100 g, 10 s) to collect the infranatant containing the SVF. The lower phase was centrifuged (400 g, 10 min), and the pellet containing the SVF was incubated for 10 min in erythrocyte-lysis buffer (155 mM NH_4_Cl, 5.7 mM KH_2_PO_4_, and 0.1 mM EDTA), filtered through 40 µm sieves, and centrifuged again (400 g, 10 min). The pellet was then resuspended in PBS containing 2 mM EDTA and 0.5% bovine serum albumin. The total number of cells was counted using Trypan blue (Gibco, Courbevoie, France) and a Neubauer hematocytometer (Poly Labo, Paul Block & Cie, Strasbourg, France). The cell count was confirmed by DNA determination using fluorometric assay (Picogreen, Invitrogen, Cergy Pontoise, France). We incubated 100,000 cells of the SVF with FITC-conjugated antibodies (F4/80), PerCP-conjugated antibody (CD45), PE-Cy7-conjugated antibody (CD11b), and respective isoptype control. Analyses were performed using a FACSCanto flow cytometer and the BD FACS Diva software (BD Bioscience). The total number of each cell population present in the fat depot was calculated as a product of the percentage of each cell type determined by the flow cytometry analyses and the total number of SVF cells. Results were presented per milligram of WAT.

In vitro triolein and cholesterol oleate hydrolase activities were performed as previously described [2]. Briefly, total protein from gonadal WAT were extracted and mixed with ^14^C-labelled triglyceride or cholesterol ester analogue, and subsequent radiolabelled fatty acids were extracted and counted. In order to estimate the enzymatic activity resulting from other lipases than HSL in these assays, the HSL-specific inhibitor was used at 1 µM [11].

For gene expression analysis, WAT was homogenized in Qiazol buffer (Qiagen) using Precellys tissue homogenizer. Total RNA from WAT was extracted using RNeasy kit (Qiagen). RNA concentration and purity were assessed spectrophotometrically using NanoDrop (DigitalBio). After treatment with DNase I (Invitrogen) and reverse transcription of 1 µg of total RNA with Superscript II (Invitrogen) or Multiscribe Reverse Transcriptase (Applied Biosystems), real-time quantitative PCR was performed with Applied Biosystems Step One Plus real-time PCR system. A standard curve was obtained using serial dilutions of WAT cDNA prior to mRNA quantitation. 18s rRNA and HPRT mRNA were used as controls to normalize gene expression.

For neutral lipid measurement, small pieces of tissues were homogenized in 1 ml 5 mM EGTA water∶Methanol (1∶2) using Precellys tissue homogenizer. Lipids were extracted with a mixture Methanol/Chloroform/Water (2.5/2.5/1.7 volume) purified with SPE column, dried, and dissolved in ethyl acetate. The fraction was measured by gas chromatography.

For glycogen measurement, liver and hind limb muscles were dissected from mice either fasted for 24 h or fasted and refed for 18 h and snap frozen in liquid nitrogen. Tissues were then dissolved in 200 µl of 1 N NaOH 1 h at 55°C. Digestion is neutralized by 200 µl of 1 N HCl and centrifuged to remove cell debris. Amyloglucosidase (50 U/ml) is then added and incubated for 1 h at 55°C. Glucose content is then measured by the RTU kit method (Biomerieux, France). Results are normalized by mg of tissue.

To determine ex vivo glucose oxidation, *soleus* skeletal muscles were homogenized with a polytron homogenizer in a buffer containing 0.25 M Sucrose, 1 mM EDTA, 1 µM Tris-HCl, and 2 mM ATP at pH 7.4. We incubated 80 µl of homogenized sample at 37°C for 2 h with 0.2 mM cold D-glucose, 1 µCi/ml [U-^14^C]glucose, 0.5% BSA, 125 mM Sucrose, 25 mM Potassium phosphate monobasic, 200 mM Potassium chloride, 2.5 mM Magnesium chloride, 2.5 mM L-Carnitine, 0.25 mM Malic acid, 20 mM Tris-HCl, 2.5 mM DTT, 0.25 mM NAD+, 4 mM ATP, and 0.125 mM Coenzyme A. Following incubation, 40 µl of 70% perchloric acid was added to trap CO_2_ production for 1 additional hour at room temperature. We counted 200 µl of NaOH, containing trapped CO_2_, using a scintillation counter (Tri-Carb2100TR; Pakard). An acidified portion was collected, placed at 4°C overnight, centrifuged at 15,000 g for 15 min at 4°C, and the supernatant was counted. A sample of the incubation medium was used to quantify specific activity.

To measure ex vivo glucose-stimulated insulin secretion by pancreatic islets, WT and HSL^+/−^ mice fed a HFD for 12 wk were anesthetized by ip injection of 50 mg/kg of pentobarbital. The common bile duct was catheterized and pancreas infused with a pre-oxygenated fixative solution [2 mg/ml of liberase (Roche) in Hanks–Hepes buffer]. Mice were sacrificed, and the pancreas was removed and digested for 8 min at 37°C. Digestion was stopped by addition of Hanks buffer supplemented with 2% BSA. After rinsing, the pellet was dissolved in 20 ml of Histopaque (Sigma) and 20 ml of Hanks/BSA buffer before being centrifuged 20 min at 1,000 g at room temperature. Pancreatic islets were collected by aspiration at the interphase. After purification, pancreatic islets were selected according to morphology and size and incubated three per well in a ACROPREP 96-well filter plate (VWR, France) in 200 µl of a medium containing 2.8 or 16.7 mM of glucose for 90 min at 37°C in an incubator. Insulin was assessed in the medium after centrifugation of the filter plate.

### Culture, siRNA Transfection, Metabolic Measurements, and Gene Expression Analysis in hMADS Adipocytes

hMADS cells were maintained in proliferation medium (Dulbecco's modified Eagle's medium low glucose, 10% fetal bovine serum, 2 mM L-glutamine, 10 mM Hepes buffer, 50 units/ml of penicillin, 50 µg/ml of streptomycin, supplemented with 2.5 ng/ml of human fibroblast growth factor 2). The cells were inoculated in 100 mm dishes at a density of 525,000 cells and kept at 37°C in 5% CO2. Six days postseeding, fibroblast growth factor 2 was removed from proliferation medium. On the next day (day 0), the cells were incubated in differentiation medium (DM: serum-free proliferation medium/Ham's F-12 medium containing 10 µg/ml of transferrin, 5 µg/ml of insulin, 0.2 nM triiodothyronine, 100 µM 3-isobutyl-1-methylxanthine, 1 µM dexamethasone, and 100 nM rosiglitazone). At day 3, dexamethasone and 3-isobutyl-1-methylxanthine were omitted from DM, and at day 10, rosiglitazone was also omitted. For glucose metabolism experiments, insulin concentration was 10 nM from day 7. The experiments were carried out between days 12 and 15.

RNA interference was achieved by small interfering RNA. Briefly, on day 7 of differentiation, hMADS cells were detached from culture dishes with trypsin/EDTA (Invitrogen) and counted. Control siGFP or gene-specific small interfering RNAs for HSL (Applied Biosystems) were delivered into adipocytes with a microporator (Invitrogen) with the following parameters: 1,100 V, 20 ms, 1 pulse. The targeted sequences, flanked with dTdT overhangs, are: GFP, 5′-GCAGCACGACUUCUUCAAG-3′; HSL, 5-AGGACAACAUAGCCUUCUU-3′.

Human fibroblast growth factor 2, insulin, triiodothyronine, transferrin, 3-isobutyl-1-methylxanthine, and dexamethasone were from Sigma; L-glutamine, penicillin, and streptomycin from Invitrogen; Hepes, Dulbecco's modified Eagle medium low glucose, and Ham's F-12 medium from Lonza; and rosiglitazone from Alexis Biochemicals.

To determine FA oxidation, cells were incubated during 3 h in 1 ml Krebs Ringer Buffer containing 3% BSA, 1 mM L-Carnitine, 80 µM oleic acid, and 20 µM [1-^14^C] oleic acid (PerkinElmer). For medium-trapped ^14^CO_2_ extraction, medium was transferred and acidified with 1 M sulfuric acid in closed vials containing a central well filled with benzethonium hydroxide. After 2 h incubation, trapped ^14^CO_2_ was measured by liquid scintillation counting. Cells were washed twice with PBS and then scraped in cold buffer (0.25 M sucrose; 10 mM Tris HCl; 1 mM EDTA; 1 mM dithiothreitol, pH 7.4). Neutral lipids and aqueous soluble metabolites were separated by adding 5 volumes chloroform/methanol (2∶1) and 0.4 volume 1 M KCl/HCl. Acid soluble products, ^14^C-labeled oxidative intermediates, were measured in aqueous phase by liquid scintillation counting. Specific activity was measured and used to calculate total oxidation as equivalent of oxidized oleic acid.

Glucose uptake was measured using 2-deoxy-D-glucose. The day before the assay, insulin was removed from culture medium. After two washes with PBS, cells were incubated 50 min at 37°C with or without 100 nM insulin. Then, 125 µM 2-deoxy-D-glucose and 0.4 µCi 2-deoxy-D-[^3^H] glucose per well were added for 10 min incubation. Culture plates were put on ice and rinsed with 10 mM glucose in ice-cold PBS and then with ice-cold PBS. Cells were scraped in 0.05 N NaOH, and 2-deoxy-D-glucose uptake was measured by liquid scintillation counting of cell lysate.

To determine glucose oxidation, insulin was removed from culture medium the day before the assay. Cells were incubated for 3 h in Krebs Ringer buffer supplemented with 2% BSA, 10 mM HEPES, 2 mM glucose, and 1 µCi D-[^14^C(U)]glucose (PerkinElmer) with or without 100 nM insulin. A 2×2 cm Whatman 3M paper was placed on top of each well and wet with 100 µl 1 N NaOH. After incubation, filter-trapped ^14^CO_2_ was measured by liquid scintillation counting. Medium-trapped ^14^CO_2_ was measured as described previously for oleate oxidation. Specific activity was counted and used to determine the quantity of oxidized glucose equivalent.

To determine glucose carbon incorporation into fatty acid and glycerol, neutral lipids were extracted after glucose oxidation as described above. They were dried and hydrolyzed in 1 ml 0.25 N NaOH in chloroform/methanol (1∶1) for 1 h at 37°C. The solution was neutralized with 500 µl 0.5 N HCl in methanol. FA and glycerol were separated by adding 1.7 ml chloroform, 860 µl water, and 1 ml chloroform/methanol (2∶1). Incorporation of ^14^C into glycerol and FA was measured by liquid scintillation counting of upper and lower phases, respectively. Specific activity was counted and used to determine the quantity of incorporated glucose equivalent.

Results from metabolic measurements were normalized to total protein content of cell extracts and expressed relative to siGFP condition without insulin.

DNA microarray and reverse transcription-quantitative PCR analysis were performed as previously described [47].

### Clinical Studies

The first clinical study cohort was comprised of 295 women and 72 men aged 18–65 years (mean 38 years), with a BMI range of 19–63 kg/m^2^ (mean 35 kg/m^2^). Thirty-eight were treated for type 2 diabetes, hypertension, and/or dyslipidemia. The remaining subjects were healthy according to self-report. They came to the laboratory in the morning after an overnight fast. A venous blood sample was obtained for the determination of fasting plasma glucose and insulin [48], which were used to calculate HOMA-IR. Thereafter, a subcutaneous fat biopsy was obtained from the abdominal area using a needle biopsy technique [49]. In 97 women and 29 men, an intravenous insulin tolerance test was performed immediately after the biopsy in order to directly estimate insulin sensitivity [48]. In the second clinical study, 25 obese individuals were investigated before and 2 years after bariatric surgery with gastric banding. At the time of the first investigation, none had undergone a slimming attempt for at least 1 year and body weight had been stable for at least 3 mo according to self-report. The third cohort, described in detail in [50], comprised 56 healthy women (age 23–72 years; mean 43 years) with a large interindividual variation in BMI (20–53 kg/m^2^; mean 33 kg/m^2^). They were investigated in the morning after an overnight fast as for the first cohort above. DNA microarray and reverse transcription-quantitative PCR analyses were performed in 45 individuals as previously described [50]. In the fourth clinical study, 24 obese men (mean BMI 32.7, [29.3; 36.5]) were randomly assigned to two groups and received placebo or nicotinic acid for 8 wk. Nicotinic acid was administered as Niaspan LP in progressive doses, reaching 2,000 mg from the fifth week to the end of the period. The study was registered in Clinical Trials NCT01083329 and EudraCT 2009-012124-85. A needle biopsy of subcutaneous WAT was performed before and after the treatment. Adipocytes were isolated by collagenase digestion for total RNA preparation. Fat cell de novo lipogenesis gene expression was determined using reverse transcription-quantitative PCR with microfluidic qPCR device (Biomark Dynamic Array, Fluidigm) [51].

### In Vivo and In Vitro Measurement of WAT Lipolysis

In mouse studies, in vitro lipolysis on isolated adipocytes was performed as previously reported [44]. In human studies, WAT was brought to the laboratory and was immediately subjected to investigation. One portion of the tissue was used for incubation in vitro exactly as described [48]. At the end of the incubation, an aliquot was removed for analysis of glycerol levels as lipolysis index. After incubation, lipids were extracted from the lWAT. In a parallel sample, isolated fat cells were prepared and mean fat cell weight determined [48]. Glycerol release was related to number of fat cells incubated by dividing total lipid weight of the sample with the mean lipid weight of a fat cell.

### Statistical Analyses

Results were expressed as mean ± SEM. Student's *t* tests, nonparametric Mann Whitney and Wilcoxon tests, and repeated measures ANOVA were used for comparisons between WT and HSL^+/−^ mice. When experiments also involved HSL^−/−^ mice, one-way ANOVA with Welch correction test was applied. Simple and multiple linear regression analyses and ANCOVA were used to analyze clinical data. Differences were considered significant for *p<*0.05 (*), *p<*0.01 (**), and *p<*0.001 (***).

## Supporting Information

Figure S1 Lipolysis in chow diet-fed HSL^+/−^ and WT mice. Mice were provided *ad libitum* access to a standard diet for 12 wk after weaning. (A) mRNA expression of HSL (lipe), ATGL (pnpla2), and plin1 in epidydimal WAT. (B–C) In vitro hydrolase activities against a cholesterol ester (B) and a TG (C) were determined in WAT homogenates in the presence and absence of a HSL-specific inhibitor to calculate the activities due to HSL. (D) Body weight curve. (E) Fat mass expressed as percentage of body weight. Values are means ± SEM. Wild type mice (▪) (*n* = 6–10) and HSL^+/−^ mice (□) (*n* = 6–10). * *p*<0.05 versus WT mice.
(PDF)

Figure S2 Gene expression in WAT of 12-wk HFD-fed HSL^+/−^ and WT mice. mRNA gene expression assessed by qRT PCR. Values are means ± SEM. WT mice (▪) (*n* = 8) and HSL^+/−^ mice (□) (*n* = 8).
(PDF)

Figure S3 Insulin tolerance test in chow and high fructose diet-fed WT mice. Mice were provided *ad libitum* access to standard or fructose-enriched diet for 45 wk. AUC, area under the curve in arbitrary unit. Body weight at the time of insulin tolerance test is presented below the curve. High fructose diet-fed mice (▪) (*n* = 7) and chow diet-fed mice (□) (*n* = 5). * *p*<0.05 versus chow diet-fed mice.
(PDF)

Figure S4 Pancreatic function in 12-wk HFD-fed HSL^+/−^ and WT mice. (A) Blood insulin during an arginine tolerance test. Values are means ± SEM. WT mice (▪) (*n* = 8) and HSL^+/−^ mice (□) (*n* = 9). (B) In vitro glucose-stimulated insulin secretion in pancreatic islets. Values are means ± SEM. The islets of three mice from each genotype have been pooled. Incubations and measurements were performed in quadruplicates. WT mice (▪) (*n* = 4) and HSL^+/−^ mice (□) (*n* = 4).
(PDF)

Figure S5 Retinol binding protein 4 measurement in media from adipocytes with HSL knockdown. RBP4 was measured in media of hMADS cells transfected with GFP or HSL siRNA. siGFP adipocytes (▪) and siHSL adipocytes (▴) (*n* = 11). ** *p*<0.01 versus siGFP.
(PDF)

## Abbreviations

ATGL: adipose triglyceride lipase
BMI: body mass index
ChREBP: carbohydrate responsive element-binding protein
DG: diacylglycerol
FA: fatty acid
GFP: green fluorescent protein
GLUT4: glucose transporter 4
HFD: high fat diet
hMADS: human multipotent adipose-derived stem cells
HSL: hormone-sensitive lipase
HOMA-IR: homeostatic model assessment–insulin resistance
NEFA: nonesterified fatty acid
QUICKI: quantitative insulin sensitivity check index
RBP4: retinol binding protein 4
TG: triacylglycerol
WAT: white adipose tissue
WT: wild type
