# Supplementary figures and images for: Partial Inhibition of Adipose Tissue Lipolysis Improves Glucose Metabolism and Insulin Sensitivity Without Alteration of Fat Mass
Source: PLoS Biol. 2013 Feb 19;11(2):e1001485. doi: 10.1371/journal.pbio.1001485 (PMC3576369; doi:10.1371/journal.pbio.1001485)

## SUPPLEMENTAL FIGURE 1

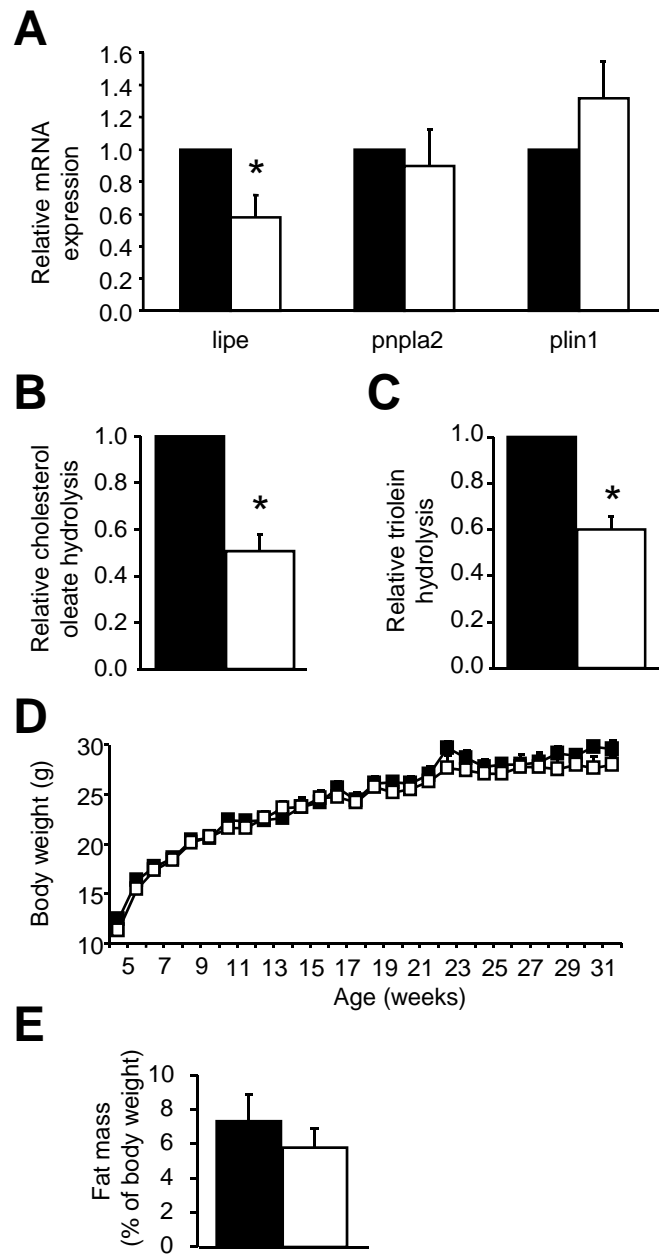

Supplement: Figure S1 — Lipolysis in chow diet-fed HSL+/− and WT mice. Mice were provided ad libitum access to a standard diet for 12 wk after weaning. (A) mRNA expression of HSL (lipe), ATGL (pnpla2), and plin1 in epidydimal WAT. (B–C) In vitro hydrolase activities against a cholesterol ester (B) and a TG (C) were determined in WAT homogenates in the presence and absence of a HSL-specific inhibitor to calculate the activities due to HSL. (D) Body weight curve. (E) Fat mass expressed as percentage of body weight. Values are means ± SEM. Wild type mice (▪) (n = 6–10) and HSL+/− mice (□) (n = 6–10). * p<0.05 versus WT mice. (PDF) [file pbio.1001485.s001.pdf]

## SUPPLEMENTAL FIGURE 2

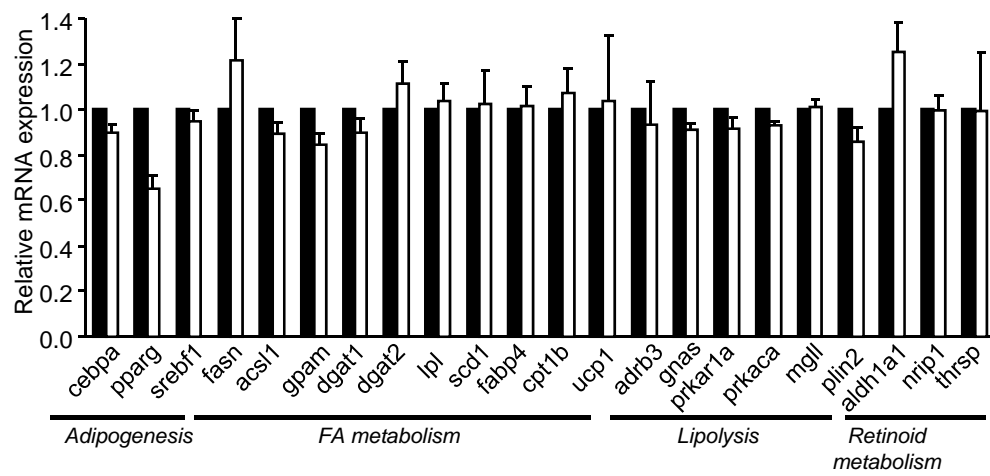

Supplement: Figure S2 — Gene expression in WAT of 12-wk HFD-fed HSL+/− and WT mice. mRNA gene expression assessed by qRT PCR. Values are means ± SEM. WT mice (▪) (n = 8) and HSL+/− mice (□) (n = 8). (PDF) [file pbio.1001485.s002.pdf]

## SUPPLEMENTAL FIGURE 3

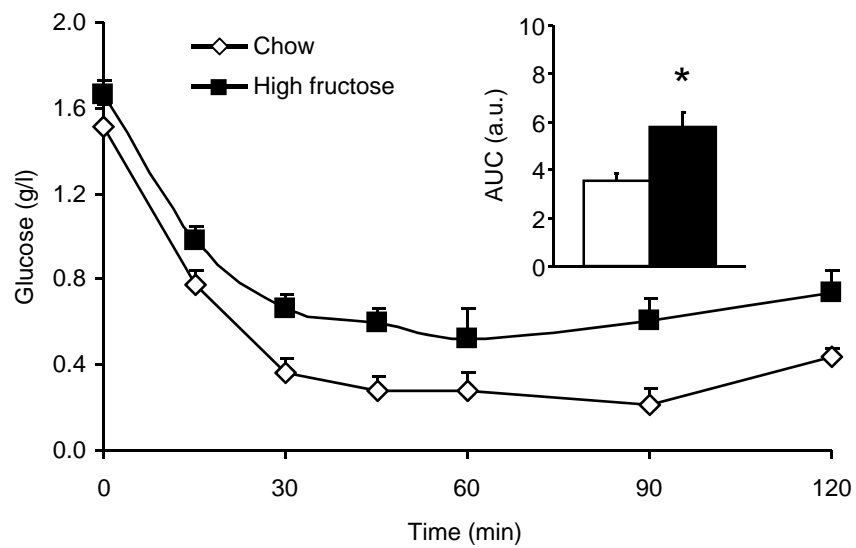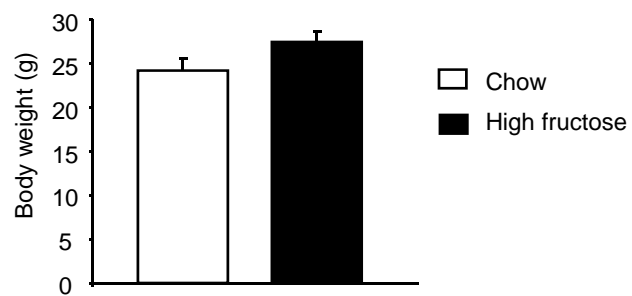

Supplement: Figure S3 — Insulin tolerance test in chow and high fructose diet-fed WT mice. Mice were provided ad libitum access to standard or fructose-enriched diet for 45 wk. AUC, area under the curve in arbitrary unit. Body weight at the time of insulin tolerance test is presented below the curve. High fructose diet-fed mice (▪) (n = 7) and chow diet-fed mice (□) (n = 5). * p<0.05 versus chow diet-fed mice. (PDF) [file pbio.1001485.s003.pdf]

## SUPPLEMENTAL FIGURE 4

**A**

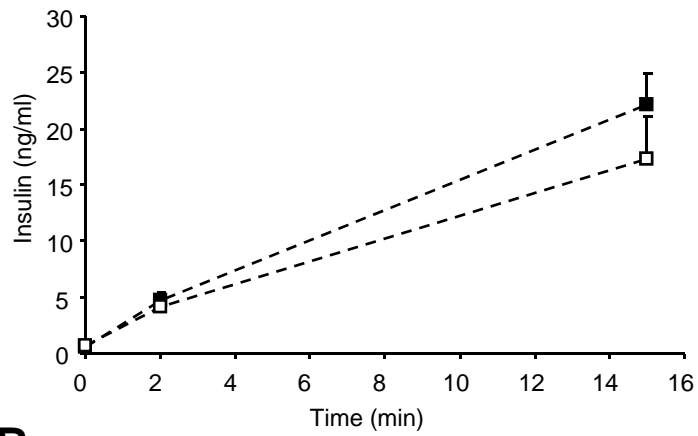

**B**

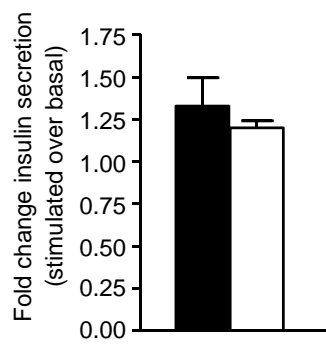

Supplement: Figure S4 — Pancreatic function in 12-wk HFD-fed HSL+/− and WT mice. (A) Blood insulin during an arginine tolerance test. Values are means ± SEM. WT mice (▪) (n = 8) and HSL+/− mice (□) (n = 9). (B) In vitro glucose-stimulated insulin secretion in pancreatic islets. Values are means ± SEM. The islets of three mice from each genotype have been pooled. Incubations and measurements were performed in quadruplicates. WT mice (▪) (n = 4) and HSL+/− mice (□) (n = 4). (PDF) [file pbio.1001485.s004.pdf]

## SUPPLEMENTAL FIGURE 5

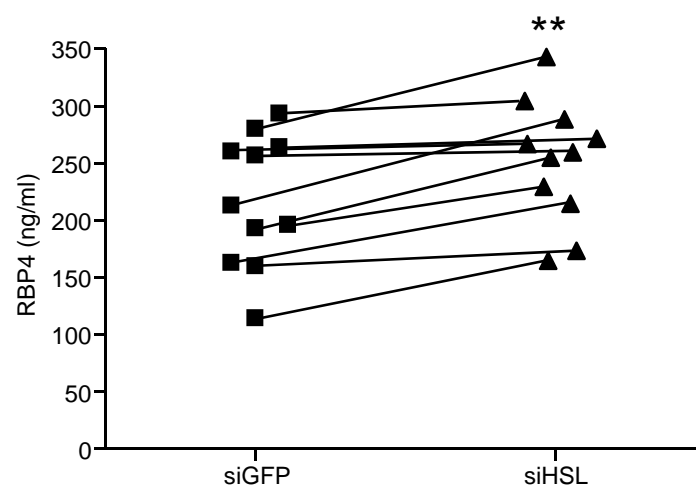

Supplement: Figure S5 — Retinol binding protein 4 measurement in media from adipocytes with HSL knockdown. RBP4 was measured in media of hMADS cells transfected with GFP or HSL siRNA. siGFP adipocytes (▪) and siHSL adipocytes (▴) (n = 11). ** p<0.01 versus siGFP. (PDF) [file pbio.1001485.s005.pdf]
